# Artists on the edge of the world: An integrated approach to the study of Magdalenian engraved stone plaquettes from Jersey (Channel Islands)

**DOI:** 10.1371/journal.pone.0236875

**Published:** 2020-08-19

**Authors:** Silvia M. Bello, Edward Blinkhorn, Andrew Needham, Martin Bates, Sarah Duffy, Aimée Little, Matt Pope, Beccy Scott, Andrew Shaw, Mark D. Welch, Tim Kinnaird, Lisa Millar, Ruth Robinson, Chantal Conneller

**Affiliations:** 1 Department of Earth Sciences, The Natural History Museum, London, United Kingdom; 2 Institute of Archaeology, University College London, London, United Kingdom; 3 Department of Archaeology, University of York, York, United Kingdom; 4 School of Environment, Archaeology, History and Anthropology, University of Wales Trinity Saint David, Lampeter, United Kingdom; 5 Department of Archaeology, Classics and Egyptology, University of Liverpool, Liverpool, United Kingdom; 6 British Museum, London, United Kingdom; 7 Wessex Archaeology, Salisbury, United Kingdom; 8 School of Earth and Environmental Sciences, University of St Andrews, St Andrews, United Kingdom; 9 School of History, Classics and Archaeology, Newcastle University, Newcastle upon Tyne, United Kingdom; Max Planck Institute for the Science of Human History, GERMANY

## Abstract

The Upper Palaeolithic is characterised by the appearance of iconographic expressions most often depicting animals, including anthropomorphic forms, and geometric signs. The Late Upper Palaeolithic Magdalenian saw a flourishing of such depictions, encompassing cave art, engraving of stone, bone and antler blanks and decoration of tools and weapons. Though Magdalenian settlement exists as far northwest as Britain, there is a limited range of art known from this region, possibly associated with only fleeting occupation of Britain during this period. Stone plaquettes, flat fragments of stone engraved on at least one surface, have been found in large quantities at numerous sites spanning the temporal and geographical spread of the Magdalenian, but they have been absent so far from the archaeological record of the British Isles. Between 2015 and 2018, ten fragments of stone plaquettes extensively engraved with abstract designs were uncovered at the Magdalenian site of Les Varines, Jersey, Channel Islands. In this paper, we report detailed analyses of these finds, which provide new evidence for technologies of abstract mark-making, and their significance within the lives of people on the edge of the Magdalenian world. These engraved stone fragments represent important, rare evidence of artistic expression in what is the far northern and western range of the Magdalenian and add new insight to the wider significance of dynamic practices of artistic expression during the Upper Palaeolithic.

## Introduction

The appearance of portable art objects during the Upper Palaeolithic is characterised by a combination of a wide choice of techniques, use of different materials and a diversity of iconographic expression. Mobiliary art perceptibly reached its prime during the Magdalenian (ca. 21,000–14,000 years cal BP) [1–4], with the development of rich decorative forms depicting animals [e.g. 5–8] or geometric designs [e.g. 9–12]. Bone and antler were extensively used to make hammers, barbed points, harpoons, needles, perforated batons (*bâtons percés*), as well as providing material for decorative forms including engravings [e.g. 4,13,14].

Stone plaquettes make up a significant proportion of Magdalenian mobiliary art [e.g. 15–21]. Plaquettes are flat pieces of stone used as a support for engraving on at least one surface. They are rarely larger than 300mm in maximum dimension and common materials used include sandstone, limestone and schist, though organic examples on flat bone (scapulae) are also known [22,23]. They are typically engraved with figurative animals or abstract ‘signs’, which can reflect a range of artistic skill. Painting with ochre or charcoal is not uncommon. Animal representations are dominated by horse and mammoth, with smaller numbers of bovids, deer, reindeer, saiga, wolf, bear, lion, fish bird and seal [e.g 16,24–28] and in a few instances, human representations [24,29–31]. Also very occasionally, aspects of the natural environment, such as rivers [18], or in some cases habitations [32], have been argued to have been depicted. Sporadically, patterns of anthropogenic use and wear are preserved but evidence generally supports a short use-life. They are often subject to heating as a result of their association with combustion features and are frequently recovered in fragmented condition, as a result of natural and/or anthropogenic breakage [16,22].

Plaquettes have been found in large quantities at numerous sites across the temporal and geographical spread of the Magdalenian. In France about 1100 stone plaquettes were found at Enlène cave, Ariège, France [33]. On the Iberian Peninsula, over 5000 stone plaquettes were uncovered at Parpalló cave in Spain [34,35] and over 1500 were found at the open air site of Foz do Medal Terrace in Portugal [36,37]. While many of these sites belong to the Middle Magdalenian and are found in the heartland of northern Iberia and southern France, the tradition and themes of plaquette engraving continue into the Final Magdalenian and survived the migration of human groups back into central and northern Europe. Amongst these more northerly sites, the open-air sites of Roc-La-Tour (Les Esprits site, Ardennes), with over 4700 specimens, has yielded by far the greatest number of plaquettes, although such abundance could be related to the fragmented nature of the finds [16]. At Gönnersdorf (Germany) slate slabs were collected locally and used as paving. Of these only 9% were engraved, but this still amounts to a total of around 500 engraved stone plaquettes [38]. Plaquettes are found in lower numbers in more north-westerly sites; they are almost absent amongst the classic Magdalenian sites of the Paris Basin with a single engraving of a horse, on a pebble rather than a plaquette, recovered from the site of Étiolles, Essonne [39]. Further west, they are also absent in Magdalenian contexts, though a slightly later (Azilian) collection of schist plaquettes was recently discovered at the site of Rocher de L’Impératrice, Brittany [40].

Extending to the far northwest in the British Islands, a restricted range of art objects has been discovered. Mobiliary art has long been known from Creswell Crags [41,42], Kendrick’s Cave [43] and Gough’s Cave [13,44–48]. However, stone plaquettes remain absent from the Magdalenian of the UK. This limited signature of art is perhaps consistent with what appears to be only fleeting occupation of Britain during the Magdalenian [49]. As a result, the recovery of engraved plaquettes on the northwest margins of the Magdalenian world is significant. This paper reports on the recovery of ten fragments of engraved stone plaquettes from the Magdalenian site of Les Varines, Jersey, Channel Islands.

## Location and archaeological context

**Fig 1:**
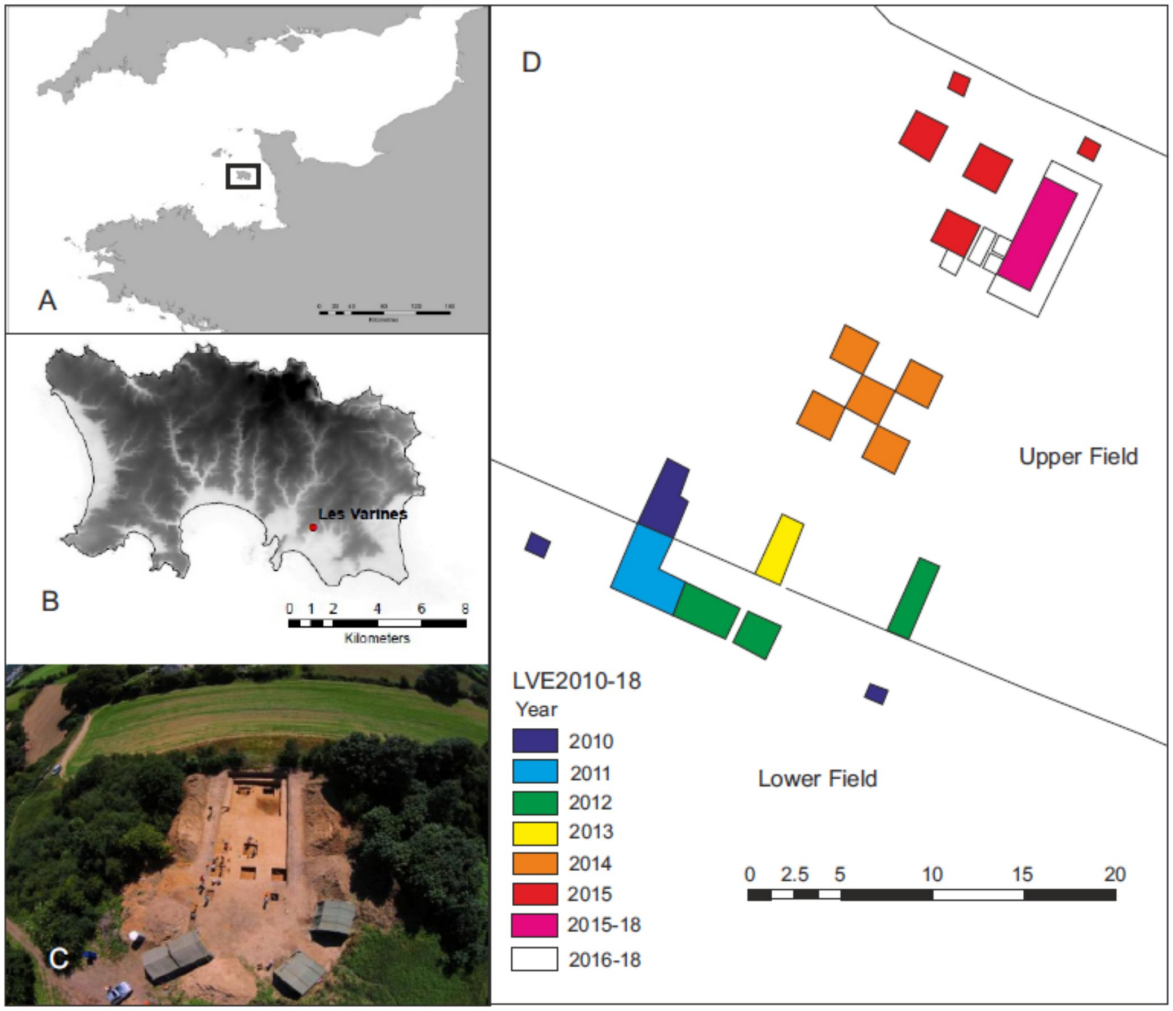
Location and archaeological context of Les Varines site. A. Location of Jersey (Channel Islands). Map data ©OpenStreetMap contributors (www.openstreetmap.org). B. Location of Les Varines site in Jersey (Reproduced under a CC BY license, with permission from Digimap (Jersey) Ltd). C. Aerial view of the site. D. Plan of excavations.

The Magdalenian site of Les Varines, St Saviours, is located on the island of Jersey, 28 km from the shores of northern France (Fig 1). The site is currently located at 35 m OD, however, during the late Pleistocene, at a time when sea-level would have been in the region of 80-100m lower, Les Varines would have been conspicuously upland in nature, commanding views far to the south over a landscape dissected by river valleys [50]. The area, which faces south, would however have been sheltered from prevailing winds. The entire island of Jersey during this time was a region of higher ground in the Manche/Channel valley, just to the south of the Channel River, a massive anastomosing system with channels up to 5 km wide. The river appears a significant barrier to the colonisation of Britain; since the initial breaching of the Straights of Dover during MIS13, almost all episodes of occupation of the British Isles in the late Pleistocene seem to have been generated by people entering Britain from the east [43,51]. Les Varines represents our only evidence of the lifeways of the pioneering groups who recolonised the southern half of Channel Valley after the last glacial maximum, at the far edge of the Magdalenian world.

Magdalenian artefacts were initially discovered as a ploughzone scatter in 2000. Fieldwork from 2010 initially focused on understanding the geological context of the site. Geophysical survey indicates the sedimentary sequence at Les Varines is complex and sediment accumulation in the vicinity of the site probably began during the Middle Pleistocene with the creation of coastal features during a high sea level event. The geomorphological context of the site is that of a cove and cliff topography in which colluvium appears to be banked against a former cliff. The Upper Palaeolithic site sits on a saddle of land between a Middle Pleistocene granite cliff and a rocky outcrop that was once a sea stack. The granite features which structure the catchment are infilled with and masked by head deposits, and constrained to the northeast and southwest by valleys.

The spread of artefacts at Les Varines extends over two fields, with the ploughzone scatter extending across part of the lower field. Results of the early seasons of excavation in this field and geophysical survey across the broader area made it increasingly obvious that the artefact-bearing sediments in the lower field have been moved downslope through periglacial processes involving the redeposition of cones of loessic silts and granitic sands. From 2014 fieldwork focused on the upper field where similar sediments were uncovered, but during 2015 intact deposits and archaeological features were encountered. These underwent further excavation between 2016 and 2018 (Fig 1D). The excavations uncovered a discreet series of head deposits, rich in lithic artefacts, spanning 50–200 mm in thickness. These deposits and the artefacts within them showed evidence for polygonal and linear modification by periglacial processes. Below this lay an apparent land surface revealing a complex spatial arrangement of hearths, granite paving, imported stones and artefactual material. Bone preservation is poor, with only a few unburnt fragments remaining; however small fragments of burnt bone are common, particularly in and around hearths where it appears to have been used as a fuel. To date, about 8500 3D recorded lithic artefacts (e.g. pieces over 10 mm) have been recovered.

**Fig 2:**
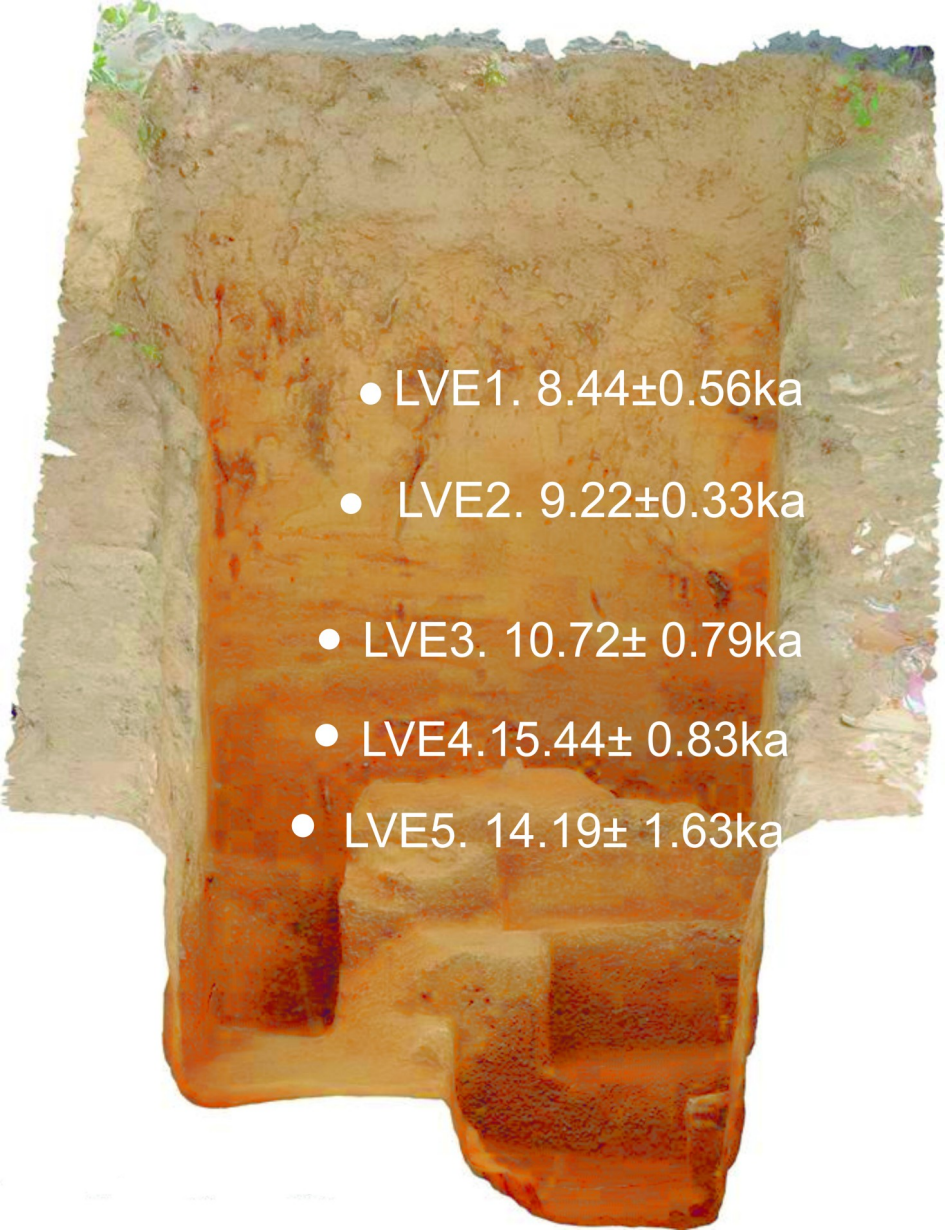
OSL-dated sequence from trench 10. Section of trench 10 (an ortho-rectified image generated using photogrammetry) showing location of OSL samples. LVE3-5 are positioned within interdigitating layers of redeposited loess [4] and granitic sand [5]. LVE1 and 2 sample yellow colluvium [3]. LVE3 and LVE5 have low numbers of aliquots and should be used only as a gauge.

An attempt to date charcoal (identified as *Betula sp*.) from one of the hearths was unsuccessful. However OSL dates are available from deposits in the lower field (Table 1). Eleven measurements were made on sequences from three trenches (for methods see S1 Appendix). All but LVE1 and LVE2 date interdigitating layers of cold climate slope deposits consisting of redeposited loess and granitic sands, several bands of which contain artefacts. The sequence from the Tr1ex is complex; while that from trench 10 (Fig 2) is more similar to elements of the sequence upslope; here redeposited Magdalenian artefacts are concentrated between LVE4 and LVE5. Though there is uncertainty and imprecision with these measurements they indicate slope deposits (containing lithic artefacts) were mobile and probably subject to numerous episodes of deposition and remobilisation probably towards the end of GS-2. They were capped by Holocene colluvium that was active into the early Holocene (LVE1 and LVE2). This sequence in trench 10 compatible with the sequence upslope in the area of hearths and pits where stratigraphic evidence indicates that occupation occurred during a time of sediment erosion and instability. This sequence in the upper field is also capped by Holocene colluvium.

**Table 1:** OSL measurements from Les Varines (Jersey).

		Radionuclide concentrations								
Sample ID	Tr.	U /ppm	Th /ppm	K /%	Water content /%	Total dose rate / mGy a^-1^	N	Stored dose / Gy	Age / ka	Dose model used
LVE 1	10	2.4 ± 0.1	9.5 ± 0.3	1.6 ± 0.1	13 ± 1	2.62 ± 0.04	52	22.1 ± 1.4	8.4 ± 0.6	MAM-3
LVE 2	10	2.3 ± 0.1	8.5 ± 0.3	1.5 ± 0.1	12 ± 1	2.51 ± 0.04	55	23.1 ± 0.7	9.2 ± 0.3	CAM
LVE 3	10	3.2 ± 0.1	12.8 ± 0.4	2.6 ± 0.1	10 ± 1	3.93 ± 0.07	7	42.1 ± 3.0	10.7 ± 0.8	only 7 aliquots—only for use as a gauge
LVE 4	10	5.3 ± 0.2	25.3 ± 0.8	3.7 ± 0.1	10 ± 1	6.16 ± 0.10	54	95.1 ± 4.9	15.4 ± 0.8	Fuch & Lang 5%
LVE 5	10	4.5 ± 0.1	22.1 ± 0.7	4.5 ± 0.1	5 ± 1	6.81 ± 0.11	1	96.6 ± 11	14.2 ± 1.6	only 1 aliquot—only for use as a gauge
LAM01	1ext	4.9 ± 0.2	22.9 ± 0.7	2.8 ± 0.1	9 ± 1	5.19 ± 0.21	34	91.2 ± 4.2	17.6 ± 1.1	CAM
LAM02	1ext	4.0 ± 0.1	21 ± 0.6	2.9 ± 0.1	6 ± 1	5.10 ± 0.21	24	54.5 ± 1.8	10.7 ± 0.6	CAM
LAM03	1ext	2.2 ± 0.1	8.5 ± 0.3	1.7 ± 0.1	12 ± 1	2.65 ± 0.10	53	38.6 ± 2.1	14.6 ± 1.0	MAM-3
LAM04	1ext	2.4 ± 0.1	8.9 ± 0.3	1.7 ± 0.1	13 ± 1	2.65 ± 0.10	53	54.2 ± 13.2	20.4 ± 5.1	MAM-3
LAM05	1ext	3.1 ± 0.1	13.4 ± 0.4	2.1 ± 0.1	13 ± 1	3.45 ± 0.13	52	61.2 ± 3.4	17.7 ± 1.2	MAM-3
LAM06	5	3.5 ± 0.1	13.7 ± 0.4	2.3 ± 0.1	4 ± 1	4.01 ± 0.17	28	58.0 ± 2.2	14.5 ± 0.8	CAM

More precision for dating the site comes from typological analysis of the lithic assemblage, which is dominated by narrow backed bladelets. Such assemblages predate the Cepoy phase of the Final Magdalenian [52], suggesting that Les Varines site is broadly contemporaneous with the classic northern Magdalenian sites of the late sixteenth and the first half of the fifteenth millennium BP such as Gönnersdorf (Germany), Pincevent and Étiolles (France). It is also potentially predates the Magdalenian of mainland Britain [53] as this lacks backed bladelets and displays chronologically later, derived features.

**Fig 3:**
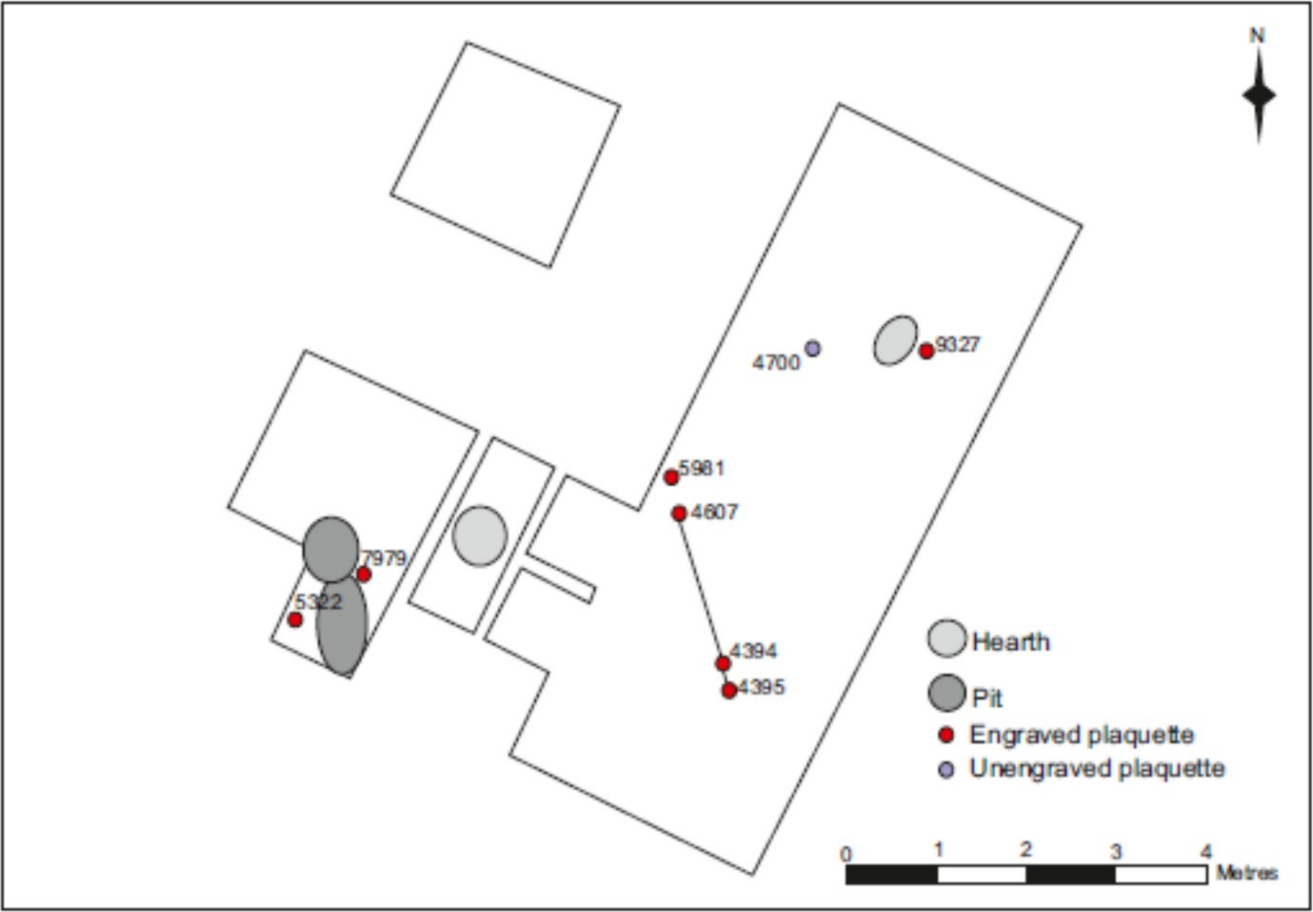
Spatial distribution of excavated plaquettes in the main area of the site (upper field).

Ten fragments of engraved fine-grained flat stones were recovered during different seasons of field excavations between 2014 and 2018 (Table 2, Fig 3). All plaquettes will be deposited with Jersey Museum, St Helier, Channel Islands. An eleventh large fragment, LVE 4700, is not engraved, but may show initial preparation for engraving. The plaquettes were recovered from three main areas:

Area 1. Fragments LVE 7979 and LVE 5322 were located just over a metre apart in the western part of the main trench in an area of pits and hearths (Fig 3).
Area 2. Three fragments (LVE 4394, LVE 4395 and LVE 4607) which refit together, were spread over an area of 2m. Fragment LVE 4607 was recovered from an area of granite slabs which may have served as paving. LVE 4394 and LVE 4395 were on the edge of the area affected by cold–weather slope movement and thus may have been affected by some post-depositional shift from their original position, which was probably originally closer to LVE 4607. Fragments LVE 9581A and LVE 9581B were found together at the end of the 2018 season, very close to LVE 4607, but at a lower level. This may represent the eastern end of the pit and hearth complex of area 1.
Area 3. LVE 9327 was also associated with a hearth, but one located in the northern part of the site, as was the possibly prepared, but unengraved piece LVE 4700 and its refitting flake.

**Table 2:** List of engraved plaquette fragments from Les Varines (Jersey).

Plaquettes	Fragment	Refit	Maximal dimensions	Year found
Plaquette 1	LVE 4394	1	29.6 x 10 x 9.5 mm	2015
Plaquette 1	LVE 4395	1	63.2 x 31.6 x 9.5 mm	2015
Plaquette 1	LVE 4607	1	85.2 x 43.9 x 8.5 mm	2015
Plaquette 2	LVE 9581 A	2	46.5 x 22.8 x 9.6 mm	2018
Plaquette 2	LVE 9581 B	2	55.9 x 36.1 x 9.0 mm	2018
	LVE 3249		87.0 x 48.0 x 16.0 mm	2014
	LVE 9327		69.1 x 68.7 x 19.7	2018
	LVE 7979		56.0 x 34.0 x15.0 mm	2017
	LVE 9458		33.4 x 31.7 x 17.2 mm	2018
	LVE 5322		28.5 x 14 x 11.5 mm	2016

The only engraved piece recovered outside these three areas was fragment LVE 3249 which was recovered from test-pit B2 to the south of the main trench, in slope-wash deposits.

## Methods

All pieces recovered were subject to macroscopic technological analysis, as well as a variety of microscopic analyses. A variety of techniques were used, focused on determining the type of stone used, tracing the incisions and determining their morphology and investigating the presence of micro-wear and residues. Refitting was also attempted to conjoin fragmented pieces. The combination of techniques used here is more broadly applicable to fragile or unique archaeological specimens.

All necessary permits were obtained for the described study, which complied with all relevant regulations. The permit to conduct archaeological excavations at the site of Les Varines (years 2010–2018) was granted by the States of Jersey (Planning Application Number P/2011/0779, P/2014/0698).

To determine the type and mineralogy of the material used for plaquette manufacture, two specimens (LVE 4607 A and LVE 4607 B), bearing incised markings, were examined by ×15 Rüper hand lens, Leica stereomicroscope and X-ray diffraction. The initial optical assessment of the rock by hand-lens as an aplite was confirmed by X-ray diffraction on a small but representative tabular chip (0.4 × 1.3 × 1.6 mm) taken from a break surface, well away from incisions. The chip was mounted on a low-background silicon wafer so that it sat flat and parallel to the surface of the wafer to allow collection in reflection mode. The wafer with sample was then attached to a goniometer head and loaded into a Rigaku-Oxford Diffraction RAPID-II DMAX X-ray diffractometer fitted with an image-plate detector and operated at 45 kV 40 mA with graphite-monochromated MoKα radiation of wavelength 0.71073 Å. This sample was then rotated using a quasi-Gandolfi movement of ϕ and ω axes in order to obtain a representative diffraction pattern that was free from orientation biases. Minerals present were identified from their characteristic signatures in the X-ray diffraction pattern using the JCPDS database.

To analyse the surface of the plaquettes for traces of manufacture and/or use, analyses were conducted both at low and high magnification [54–56]. A stereoscope ranging in magnification from 5X - 7.5X was first used to screen the artefacts for traces of wear and residue. High magnification analysis was then undertaken with a Leica DM2500 MH transmitted light microscope using 10X and 20X lenses. The engravings themselves on the different plaquette fragments were initially examined using a hand lens and binocular microscope. The incisions were further examined using an LEO1455VP and a Quanta 650FEG SEM (FEI Company) scanning electron microscope (SEM), operated in variable pressure mode (chamber pressure respectively 15 Pa and 70 Pa). The high resolution of SEM images allows better characterisation and appreciation of the incised forms by identifying the agent responsible for their production (natural vs humanly induced; the type of tool used; [57]). In order to identify the elemental composition of possible exogenous material adhering to the stone surface, Energy-dispersive X-ray spectroscopy (EDX) analysis was conducted using the FlatQuad 5060F detector (Bruker) with the following microscope setup: 10kV, spot 3.0, chamber pressure 30Pa. Standardless quantification was done with the interactive Pb-ZAF algorithm.

The topography of surface modifications was recorded using a Focus Variation Microscope (FVM), the Alicona InfiniteFocus optical surface measurement system. This system was used to produce three-dimensional (3D) micro-morphological models of the incisions according to the methodology described by Bello and co-authors [44,58,59]. The 3-dimensional reconstruction of the topography of the engraved surfaces allows a better recognition and interpretation of the technical and gestural procedures followed during engraving [9,57,60,61]), including the chronological order of the marks [28,58,62–64]. The full length of the incision (L) was measured when possible (cf. when the complete incision was preserved). Profile micro-morphometric parameters were measured at the incisions’ midpoint and consist of the following: width of the incision at the surface (WIS), width at the bottom of the incision (WIB), opening angle of the cut (OA) and depth (D) of the incision ([58], pages 2467–2469 and Fig 5 within). The incisions were differentiated into either ‘single-stroke’ incisions, obtained by a single tool passage, or ‘multiple-stroke’ incisions, when made by multiple tool passages (for more details refer to [44]).

Several computational image-based visualisation approaches were also applied during the analysis of the plaquettes in order to gain a better understanding of the design reproduced on each fragment. All fragments of stone plaquettes were photographed at the Image Suite at the Natural History Museum (London, UK), using digital camera and macro lenses. For some of the fragments we used Reflectance Transformation Imaging (RTI). This is one of the most informative of the techniques and fundamental in terms of rapidly assessing the artefacts as they progressed through post-excavation analysis in the field. Using this multi-light recording approach, per-pixel information about surface shape and colour is captured in a set of digital images and interactively visualised post-processing revealing subtle surface detail. First developed at HP Laboratories by Malzbender and Gelb [65,66], the technology has subsequently been enhanced/expanded with alternative processing and capture approaches [67–70]. For the purposes of this research, the Highlight-RTI (H-RTI) acquisition method was selected based on its flexible, non-contact nature, portable and relatively inexpensive tool kit as well as its potential to reveal information about the engravings, possibly not visible upon physical inspection.

One of the key limitations of RTI is the lack of metric data. To address this, macro Structure from Motion (SfM) Photogrammetry was also trialled on the engraved pieces. More commonly used on a larger scale in the context of excavation, the technique entails the generation of 3D geometry and models from a set of overlapping images. Although the results from the application of this technique were less than optimal in terms of the information provided about the surface topography of the plaquettes, the macro imagery produced from the trial proved useful when processed with supplemental colour filtering software called D-Stretch. Developed to reveal subtle features of painted rock art, D-Stretch enhances colour information in images with various filtering algorithms [71]. For the purposes of this research, it was used to test for the presence on pigment on engraved pieces from Les Varines. Small patches of possible pigment noted through this method underwent further testing by EDX analysis.

We produced the illustrations of the plaquettes and engraving using Adobe Illustrator software, by digitally drawing over the photos of each fragment of the stone plaquettes. This recording method was supported by continuous inspection of the original specimen and the aid of high quality RTI and Alicona images. The sequence of the engraving was determined by direct macro and microscopic analyses, particularly using Alicona micro-imaging, which allows tilting of the scanned surface at different angles, enabling the recognition of the order of superposition of the incisions. The sequence of engraving for each stone fragment has been reproduced on the drawing using colour coding.

## Results

The ten engraved stones described in this paper are fragmented flat pieces of aplite/microgranite. Fragments LVE 4394, LVE 4395 and LVE 4607 (a, b, and c) refit together in one large plaquette (plaquette 1). Fragments LVE 9581A and LVE 9581B, found in connection, refit in an almost complete circular plaquette (plaquette 2). No refits were made between the other five fragments (LVE 3249, LVE 9327, LVE 7979, LVE 9458 and LVE9 5322) (Table 2). An eleventh fragment, LVE 4700, is not engraved, but has a refitting flake which may be related to initial shaping of this material. Thirty additional stone fragments morphologically similar to the engraved pieces were also recovered from the site. These are small (generally in the region of 20–40 mm) and are not engraved, however the vast majority of these lack any trace of the flat smooth surface where engravings might be found. They may be frost fractured pieces of plaquettes or plaquette blanks, but also could conceivably be natural material eroded from an original source in the vicinity. No refitting was possible between these smaller pieces and any of the engraved specimens.

**Fig 4:**
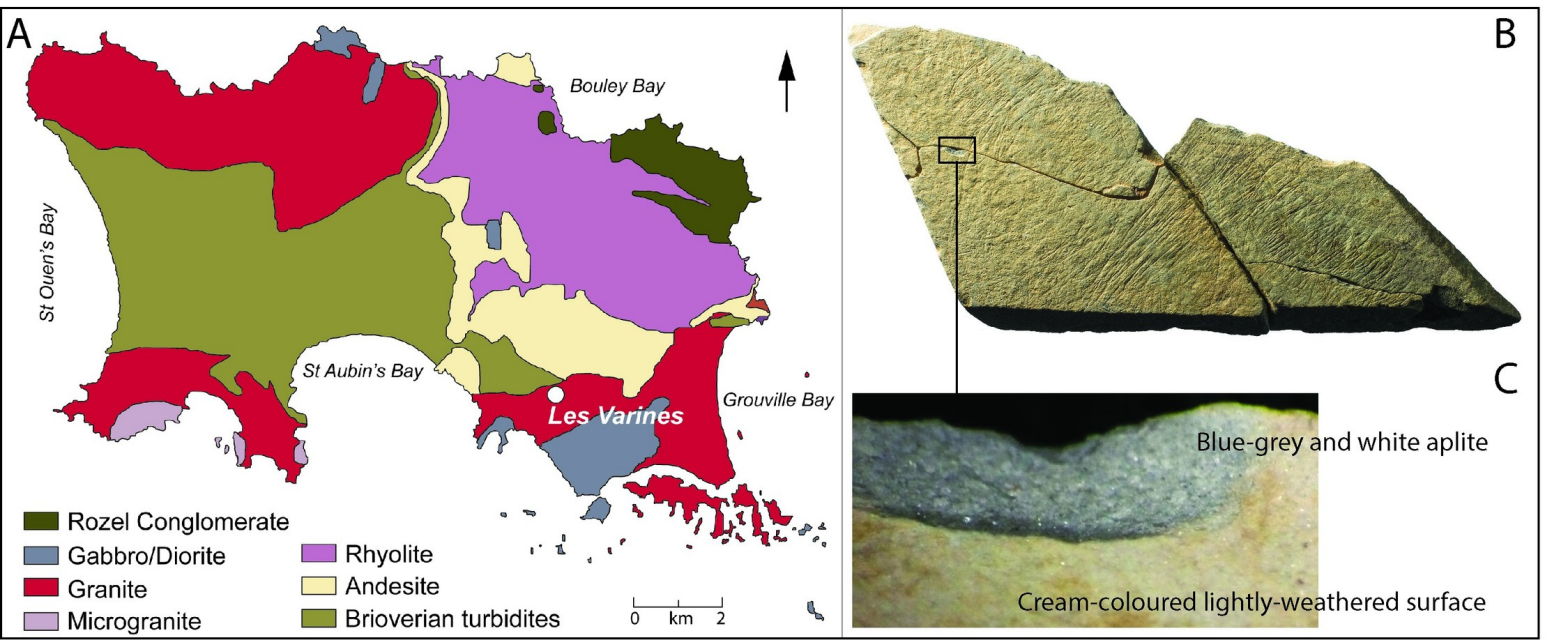
A. Geological map of Jersey (modified from Brown et al. 1990. p. 195) with location of the Magdalenian site of Les Varines. B. Photo of Plaquette 1 (LVE 4394, 4395 and 4607) and C. detail of blue-grey and white aplite rock type and lightly-weathered surface.

Specimens LVE 4607 a and b were examined in order to determine the rock type and its mineralogy. The rock is an aplite comprised predominantly of inter-grown fine-grained crystals of albitic feldspar and quartz, with very minor amounts of muscovite and biotite micas, the latter seen as black sub-millimetre clot-like aggregates dispersed sparsely throughout the aplite. Minor chlorite is also associated with biotite. The rock is texturally homogeneous and has an overall “sugary” aspect, with a thin (1mm) white lightly-weathered surface overlying pristine blue-grey and white aplite. The incised lines are seen on these surfaces, which in places have fresh aplite exposed as contrasting bluish-grey patches (Fig 4B and 4C). The strongly weathered parts of the rock are stained orange-brown by chemical alteration which is seen as Liesegang fronts. The fine-grained nature of the aplite allows incisions to be made that could not be worked on coarser granitic rocks. The geology of Jersey is dominated by ancient granites associated with the Cadomian orogeny (450–700 Ma). The incised rocks reported here are of a type (aplite/microgranite) that is common in the South East/St Helier granitic complex, and so it is probable that they were sourced locally by Magdalenian groups (Fig 4A).

**Fig 5:**
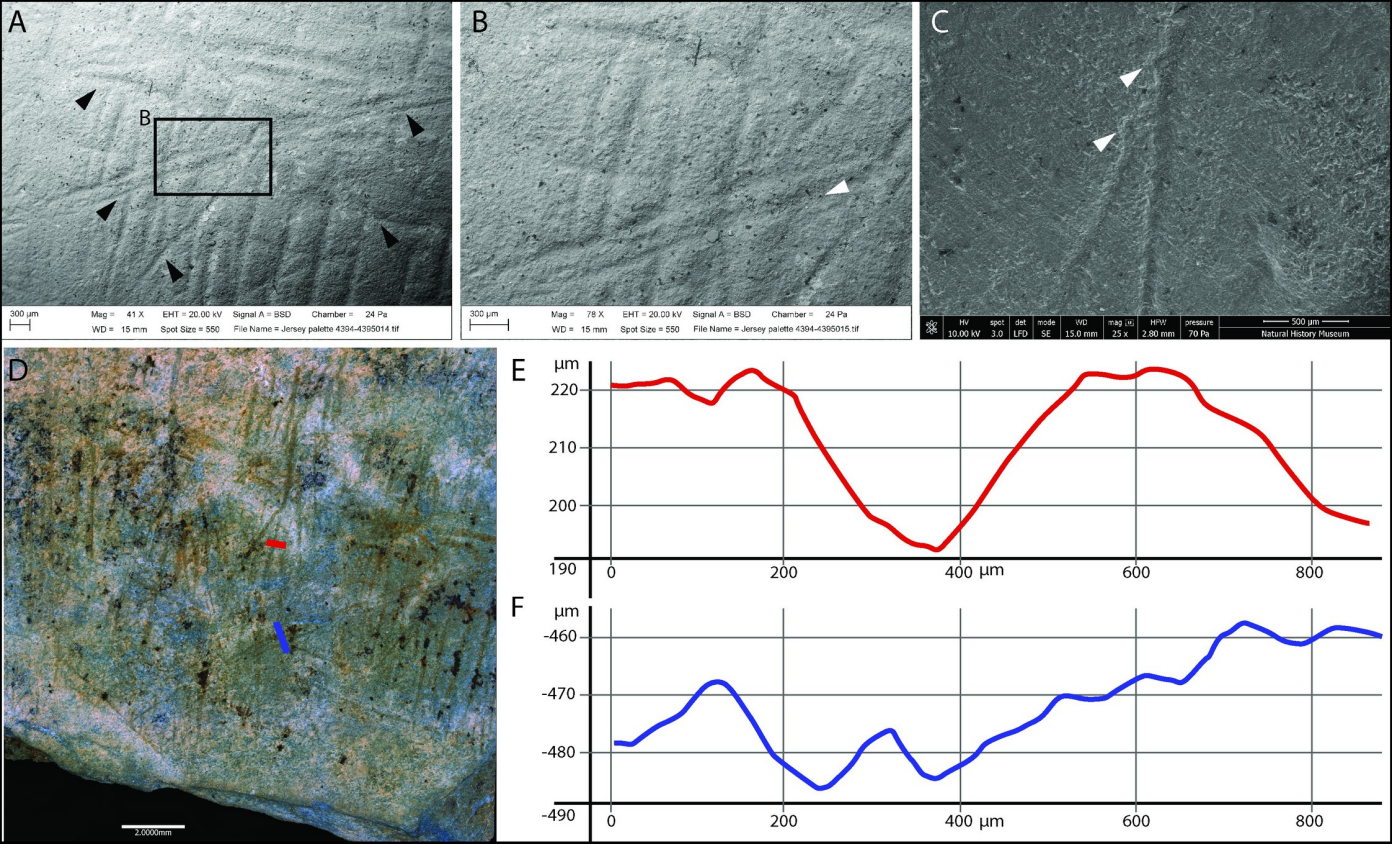
SEM images indicating features typical of incisions by a stone tool. A. overlapping of incisions produced in succession and with different orientations (black arrows). B. Internal microstriations (white arrow); C. Hertzian cones (white arrows). D. Alicona photo image of a portion of plaquette LVE 4394 and details of E. Single stroke incisions (red profile) and F. Double stroke incisions (blue profile).

SEM analyses of the engraved slabs confirm the general good state of preservation of the surfaces. No obvious trampling marks or weathering are visible on the engraved surface, however, the engraving marks themselves appear rounded and slightly polished, possibly as a consequence of gentle surface abrasion produced by periglacial processes while buried in the sediment. This was also noted in the microwear study with a generic weak polish (GWP) visible across the upper topography of most surfaces. Because of this process, the incisions do not have the v-shape with converging slopes and narrow bottom typical of incisions produced by a stone tool. However, it is still possible occasionally to observe other characteristics typical of incisions produced by stone tools [e.g. 72–80] (Fig 5A–5C).

### Plaquette 1 (LVE 4394, 4395 and 4607)

**Fig 6:**
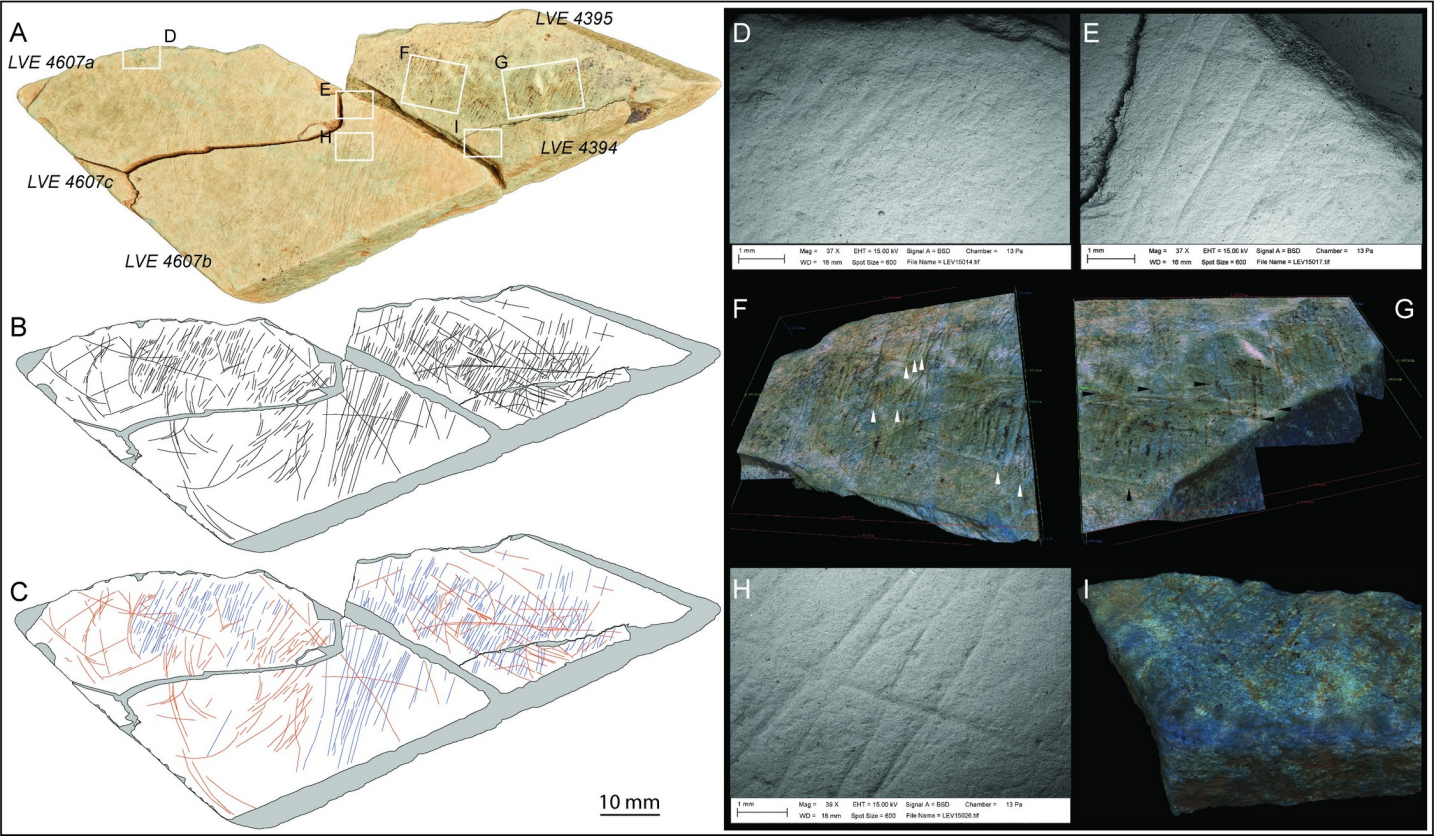
Plaquette 1. A. Photo and B. drawing of plaquette 1 (LVE 4394, 4395, 4607a, b and c). C. drawing of plaquette 1 distinguishing straight incisions (blue lines) from sinuous incisions (orange lines). SEM images of interrupted incisions at the edge of (D) LVE4607a and (E) LVE4607b. 3D Alicona images of two different areas on LVE 4395. White arrows (F) show examples of bifurcation of the final portion of some straight incisions; black arrows (D) show examples of sinuous incision overlapping straight incisions. The overlapping of sinuous incisions over straight incisions is also visible in (H) a SEM image of fragment LVE 4607b and (I) an Alicona 3D image of fragment LVE 4394.

Three fragments, LVE 4394, 4395, 4607a, b and c (Fig 6A), refit together to form part of a larger plaquette (plaquette 1). The largest of these three pieces (LVE 4607) had fractured in situ into three fragments (a, b and c), which were found still in connection in the ground and were therefore given a single inventory number. Fragments LVE 4394 and 4395 refit together, although they were found 30cm apart. LVE 4394 is a small flake that has detached from the external surface of fragment 4395. When all pieces are refitted together, plaquette 1 has a broadly triangular form and measures 120x44x9.5mm. It is the thinnest of the plaquettes and has a particularly fine, smooth surface. The plaquette, pale-green or pale-brown in colour, is a piece of aplite/microgranite.

A generic weak polish (GWP) is visible across the upper topography of plaquette 1, probably resulting from gentle abrasion with the surrounding soil matrix. The edges of the plaquette’s fragments displayed rounding and GWP, indicating that they were fragmented sometime in antiquity. The plaquettes did not display any traces which might indicate surface preparation for the engravings (e.g. grinding/smoothing); however, if such traces were present and also superficial, they may not have preserved, especially given the evidence for surface abrasion.

A series of incisions that continue across the different fragments of this palette confirm the productions of the engraving when plaquette 1 was still unbroken (Fig 6D). The presence of several interrupted incisions at the edges (Fig 6A–6E) suggest that plaquette 1 is incomplete and was larger in its original unbroken form. One smoother edge is likely to represent the natural edge of the block. Though plaquette 1 is broken, there are no unequivocal signs of intentional fracture.

Plaquette 1 is marked by two types of incisions: straight lines more or less parallel to each other and with the same orientation (blue lines in Fig 6C), which cover much of the plaquette and more sinuous lines with irregular orientation (in orange in Fig 6C). Both types are produced by a combination of (a) ‘single-stroke’ incisions, (b) ‘double’ incisions with recognizable internal sub-incisions [*sensu* [58]] produced by a stone tool engraving twice the same area. This double engraving is often associated by bifurcation at the end, suggesting the second incision was intended to extend the design beyond the first incision (Fig 6F, indicated by white arrow). In some cases, however, the ‘double-dip’ of an incision is more likely the result of breakage of the tip of a stone tool or the use of burins. The longer and curved engraved lines generally overlap, with very few exceptions, the straight lines, suggesting they were the last to have been produced (Fig 6H–6I).

Despite the two sets of engraving being produced at different times, the micro-morphometric analyses of the marks highlight the similarity in all profile parameters (width, opening angle and depth) of the two types of incisions (Table 3). This close similarity suggests that the two types of marks were produced using the same tools, probably by the same engraver and in short succession.

**Table 3:** Average micro-morphometric values for the incisions on the five fragments forming Plaquette 1.

Stone fragment	Type of Incision	No Measured		Length (mm)	WIS (μm)	WIB (μm)	OA (°)	D (μm)
LVE 4394	BLUE	0–16	Mean =		**308.5**	**98.3**	**156.7**	**25.7**
			SD =		89.7	38.5	6.7	9.9
			Min =		163.2	56.3	146.8	13.6
			Max =		489.3	193.5	168.4	49.5
	ORANGE	0–6	Mean =		**396.3**	**123.7**	**159.4**	**24.2**
			SD =		142.9	52.9	4.0	5.6
			Min =		234.3	52.5	153.9	18.1
			Max =		580.6	186.5	164.1	32.5
LVE 4395	BLUE	58–59	Mean =	**5.8**	**361.8**	**120.9**	**153.6**	**31.1**
			SD =	2.1	130.5	52.4	8.0	11.0
			Min =	1.8	168.0	49.1	132.7	8.2
			Max =	10.0	791.7	328.5	170.2	58.2
	ORANGE	26–27	Mean =	**8.7**	**344.6**	**97.0**	**154.7**	**27.6**
			SD =	3.7	135.1	50.1	10.2	14.4
			Min =	2.9	160.6	27.08	124.9	10.6
			Max =	14.8	623.9	233.8	169.1	77.0
LVE 4607	BLUE	37–37	Mean =	**6.6**	**263.3**	**82.3**	**154.6**	**21.8**
			SD =	4.0	96.6	35.0	9.3	9.3
			Min =	2.4	116.3	28.2	129.6	10.2
			Max =	16.1	576.9	187.8	168.4	49.9
	ORANGE	22–22	Mean =	7.3	310.3	106.3	154.8	24.8
			SD =	2.9	126.1	56.3	9.3	9.7
			Min =	3.2	148.1	54.5	128.7	9.9
			Max =	12.8	658.3	283.1	170.5	46.2
**Plaquette 1**	**BLUE**	**95–112**	**Mean =**	**6.1**	**321.6**	**104.9**	**154.4**	**27.2**
			**SD =**	**3.0**	**122.6**	**48.4**	**8.3**	**11.1**
			**Min =**	**1.8**	**116.3**	**28.2**	**129.6**	**8.2**
			**Max =**	**16.1**	**791.7**	**328.5**	**170.3**	**58.2**
	**ORANGE**	**48–55**	**Mean =**	**8.1**	**336.5**	**103.6**	**155.3**	**26.1**
			**SD =**	**3.4**	**132.6**	**52.7**	**9.4**	**11.9**
			**Min =**	**2.9**	**148.1**	**27.1**	**124.9**	**9.9**
			**Max =**	**14.8**	**658.3**	**283.1**	**170.5**	**77.0**

No of measured incisions: the first number refer to how many lengths of incisions were measured, the second number refer to how many time profile parameters were measured. WIS: width at the surface; WIB width at the bottom of the cut; OA: opening angle of the incision; D: depth of the incision, ATI: angle of tool inclination.

**Fig 7:**
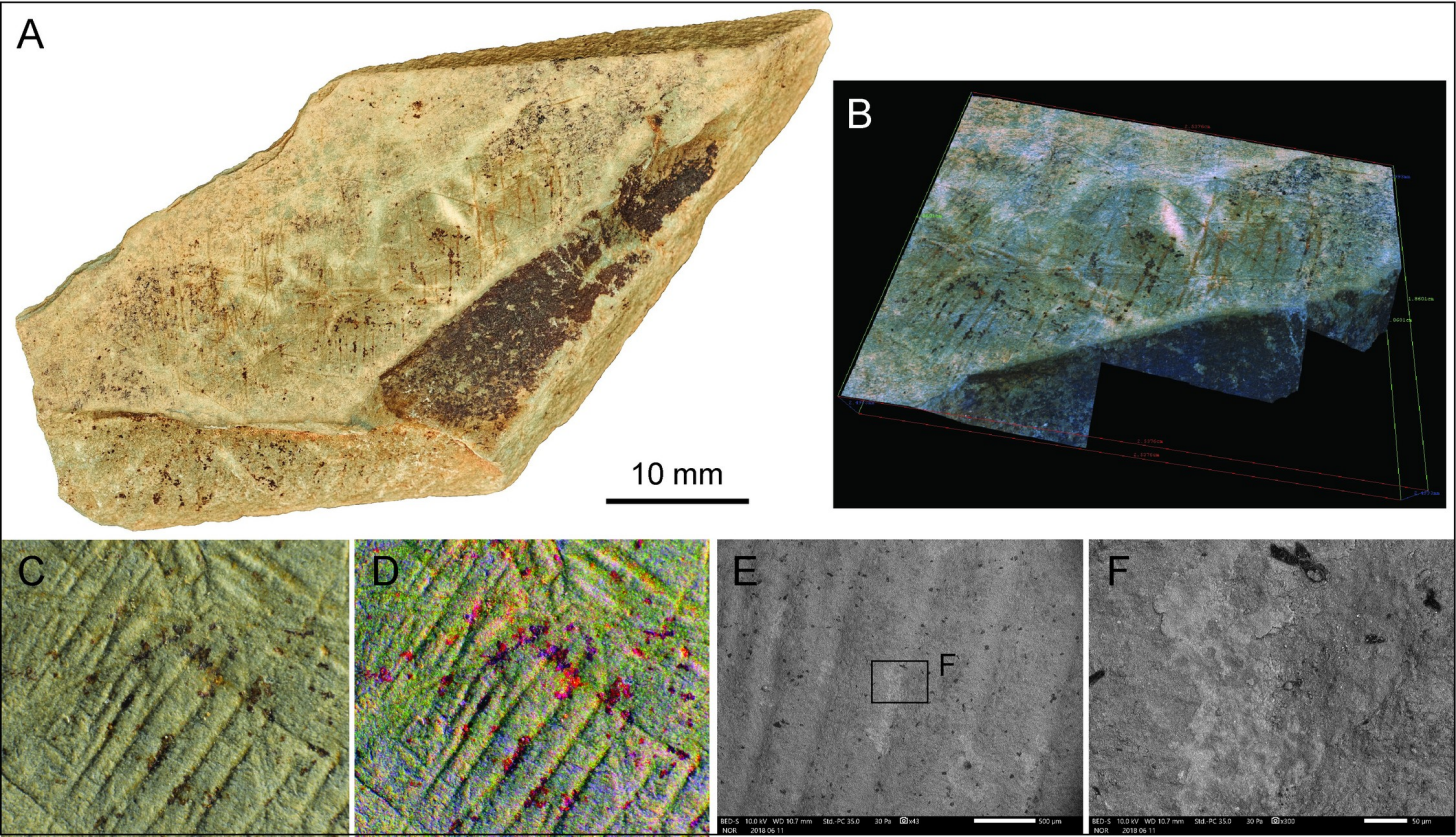
LVE 4395. A: Photo of fragment LVE 4395 showing red stain patches on the engraved surface and on the breakage surface with fragment LVE 4394. B: 3D Alicona images of patches of reddish stain on LVE 4395. C. Macro-image of stain patches from photogrammetry image set. D Impact of D-stretch filtering in highlighting stain patches. E-F SEM images of stain patches. The red patches appear in lighter (whiter) grey tone.

Some engraved marks on LVE 4395 appear ‘coated’ by minute patches of reddish stain identified initially through Structure from Motion photogrammetry and D-Stretch colour filtering as described above (Fig 7). EDX analyses could not detect any differences in the mineral composition of these patches and the surrounded surface of the plaquette. The microscopic aspect of these small patches is not dissimilar from the larger stain observed on LVE 4700 (description below). However, a similar larger stain is also present on the breakage surface on LVE 4395, along the edge that refits with LVE 4394. This stain is not present on LVE 4394. The presence of engraved lines between LVE 4395 and LVE 4395 interrupted by this breakage suggest that their separation occurred after the plaquette was engrave, while the distribution of the stain would suggest the stain of LVE 4395 occurred after the breakage of plaquette 1 in multiple fragments. It is therefore likely that this stain was not part of the decoration of plaquette 1, differently from other Magdalenian examples where ochre was used on the stone plaquettes [39].

**Fig 8:**
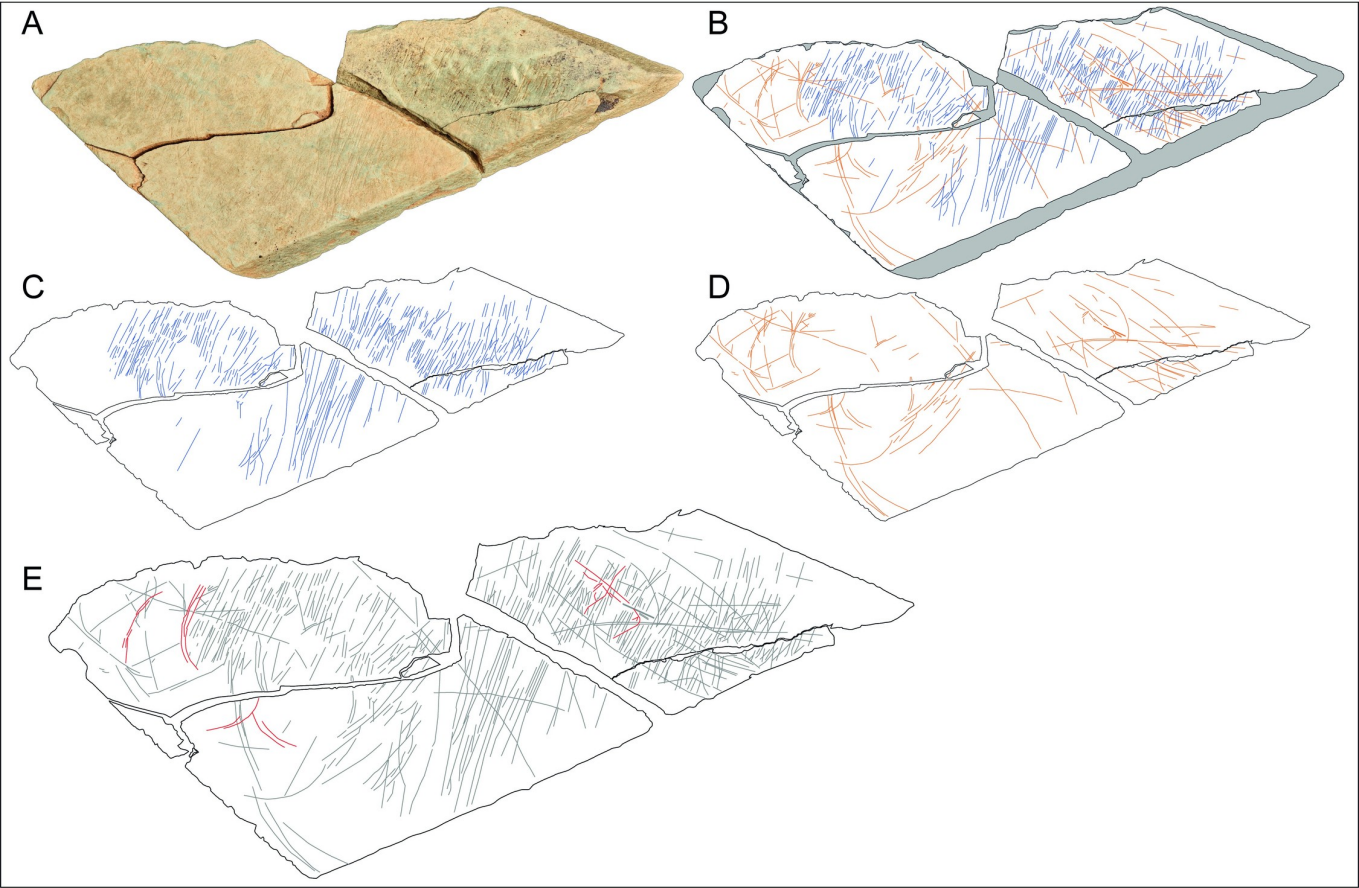
Plaquette 1. A. Photo and B. drawing of plaquette 1 (LVE 4394, 4395, 4607a, b and c). Drawings separating the sequence of engraving: C. straight lines (blue lines) and D. superimposed sinuous incisions (orange lines). E. Tentative interpretation of some possible zoomorphic features (red lines) present on plaquette 1.

The artistic design on plaquette 1 is difficult to interpret. There are over 450 intersecting incisions on the engraved surfaces of the five fragments. The parallel lines were the first to be made and are the more abundant type of design followed by ramified lines (lines ending in bifurcation usually of similar length) (Fig 8C). Parallel and ramified lines are combined and alternate in long sequence, a pattern also observed on plaquettes at Roc-La-Tour, site des Esprits (Ardennes, France; [16] Fig 16 within) and Barma-Grande (Italy; [12]) or in cave art (e.g. Altamira, Spain; [81]). The more rounded long incisions do not seem to show any clear pattern or obvious representation (Fig 8D). They overlap the straight lines, and only in few instances, they could tentatively be interpreted as the representation of zoomorphic parts: the back or the belly of horses, deers or bovids and possibly the depiction of the snout of a bovid on its right profile (Fig 8E, red lines). None of these elements, however, is detailed enough to offer an unequivocal interpretation, and is far from the carefully crafted zoomorphic representations depicted on stone plaquettes such as those from Le Rocher (Britany, France; [40]).

### Plaquette 2 (LVE 9581 A, 9581 B)

LVE 9581 A and LVE 9581 B were found in connection to form an almost complete plaquette (plaquette 2). Plaquette 2 has a broadly oval form and measures 57.5 x 55.9 x 12.7mm. The plaquette has a particularly fine, smooth surface. As with Plaquette 1, the rock comprises bluish-grey patches of fresh aplite and pale-brown areas richer in aplitic feldspar that is partially altered to kaolinite.

**Fig 9:**
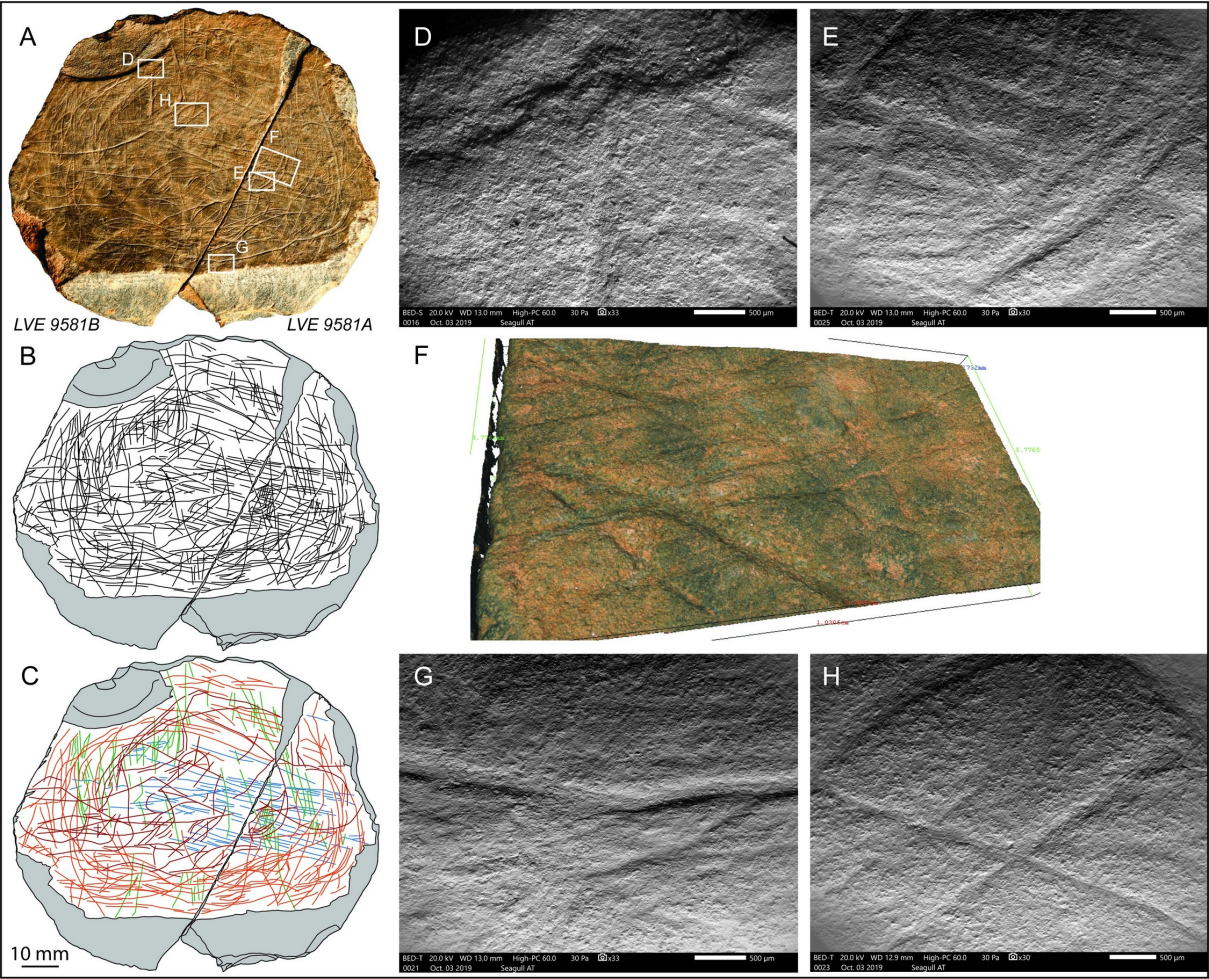
Plaquette 2. A. Photo and B. drawing of plaquette 2 (LVE 9581A and LVE 9581B) C. drawing of plaquette 1 distinguishing straight incisions (blue and green lines) from sinuous incisions (orange and red lines). D. SEM images of interrupted incisions at the edge of (D) LVE 9581 B4607A. SEM (E) and Alicona 3D image (F) detailing the complex set of overlapping incisions. G. SEM image of interruption and resumption of an incision. (H) Overlapping incision forming an encircled cross.

As with plaquette 1, several incisions continue across the two fragments of the plaquette indicating the engravings were produced when plaquette 2 was still unbroken (Fig 9). In contrast to plaquette 1, however, almost all incisions at the edges of plaquette 2 appear to be contained within one of the two fragments, the only few exceptions are associated with flaking at the edge of the plaquette (Fig 9D). This would indicate that LVE 9581 A and B, apart from few damaged edges, form an almost complete plaquette, the only one found so far at the site. Though this is an ancient break, there are no unequivocal signs of intentional fracture of plaquette 2; its close connection suggests the break may be the result of post-depositional processes.

Plaquette 2 is the most elaborately marked stone plaquette from the site with several intersecting and superimposed incisions (Fig 9). We recognise 4 types of incisions. The first marks to be made were long, straight, sub-parallel marks particularly concentrated in the central portion of the plaquette (blue lines in Fig 9C). These marks are generally narrow and shallow. They are least obviously visible and are cut across by all other types of incisions found on the plaquette. Perpendicularly to these incisions is another set of straight, sub-parallel marks (green lines in Fig 9C). These ‘green’ incisions are similar to the blue lines, as they also are generally sub-parallel to each other and are made by a single tool passage. The ‘green’ engravings are morphometrically the thinner and shallower incisions observed on plaquette 2 and may possibly represent a form of decoration or ‘shadow’ effect to the overall design. The green lines are concentrated at the edge of the stone plaquette 2, with only a few lines cutting across its central portion. In some areas both the ‘blue’ and the ‘green’ engravings appear to be interrupted at the edge of the plaquette. These rare interruptions correspond with flaking damage along the edges of the plaquette (Fig 9D), confirming the almost completeness of plaquette 2 and suggesting that the rich and complex engraved design was fully contained within the smooth surface of plaquette 2.

The majority of the incisions are sinuous lines that form a semi-circular concentric design toward the edge of the plaquette (orange lines in Fig 9C). These incisions give the impression of being wider and overall bigger than the ‘blue’ and ‘green’ lines, but this appearance is rather the results of multiple single incisions made very close to each other, as the morphometric characteristics of each single incision are overall not too different from the two sets of subparallel straight (blue and green) incisions (Table 4). The circular design they form is not continuous, but rather the result of several line traced one after the other, with clear examples of resorption and re-starting points (Fig 9G). The most irregular incision (red lines in Fig 9C) cut across the entire surface of plaquette 2. They are sometimes circular, similarly to the orange lines, sometimes sinuous. They overlap all other incisions and are morphometrically wider and deeper than the other as they were made using a combination of single and multiple stroke passages (Table 4).

**Table 4:** Average micro-morphometric values for the incisions on LVE9581 (A and B).

Stone fragment	Type of Incision	No Measured		Length (mm)	WIS (μm)	WIB (μm)	OA (°)	D (μm)
LVE 9581 (A and B)	BLUE	25/30	Mean =	**8.90**	**321.0**	**106.4**	**158.1**	**24.4**
			SD =	4.09	110.0	43.0	9.9	12.8
			Min =	4.27	127.8	49.3	130.0	6.8
			Max =	19.48	527.3	190.2	170.9	53.5
LVE 9581 (A and B)	GREEN	26/30	Mean =	**4.44**	**271.7**	**108.5**	**164.1**	**15.1**
			SD =	2.27	100.1	45.7	4.2	7.6
			Min =	1.42	121.3	35.7	153.4	3.9
			Max =	10.01	530.1	221.5	172.2	36.7
LVE 9581 (A and B)	ORANGE	34/46	Mean =	**11.05**	**330.3**	**112.6**	**158.2**	**24.4**
			SD =	4.78	112.1	35.6	8.9	10.7
			Min =	3.97	157.5	44.6	136.0	6.8
			Max =	22.11	720.2	208.5	173.7	51.0
LVE 9581 (A and B)	RED	24/39	Mean =	**9.85**	**404.2**	**137.3**	**157.7**	**32.3**
			SD =	5.73	128.2	45.9	9.3	15.1
			Min =	1.9	222.5	63.8	134.0	12.3
			Max =	25.8	935.0	321.2	171.4	77.6

No of measured incisions: the first number refer to how many lengths of incisions were measured, the second number refer to how many time profile parameters were measured. WIS: width at the surface; WIB width at the bottom of the cut; OA: opening angle of the incision; D: depth of the incision, ATI: angle of tool inclination.

**Fig 10:**
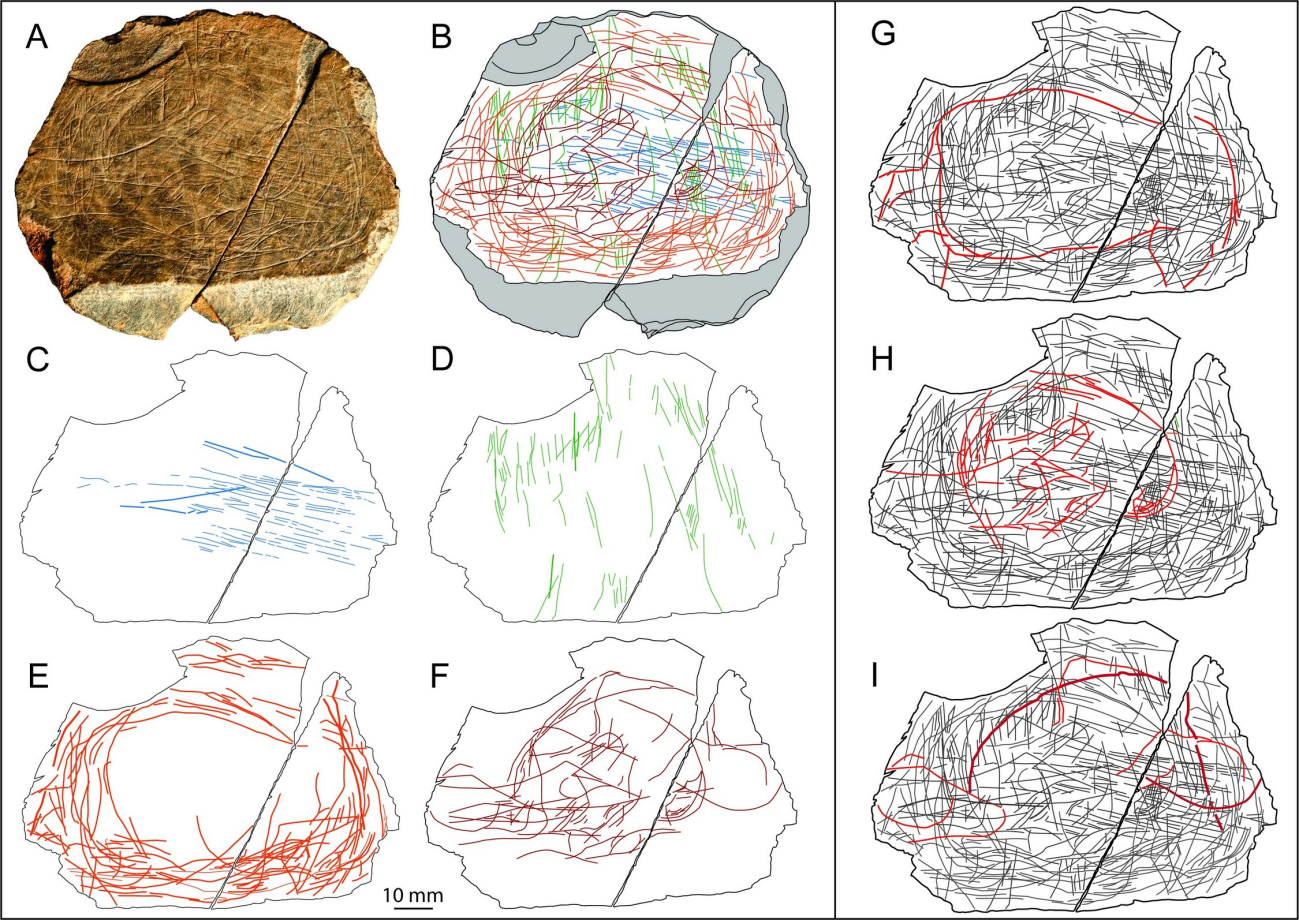
Plaquette 2. A. Photo and B. drawing of plaquette 1 (LVE 9581A and LVE 9581B). Drawings separating the sequence of engraving: C. straight lines (blue lines) and D. straight lines (green lines) perpendicular to the previous ones. E. Sinuous, semi-circular concentric incisions (orange lines) and F. sinuous large and deep incisions (red lines), the last one to have been engraved. Tentative interpretation of a possible bovid (G), a human face (H) and simplified zoomorphic features of a horse and 3 mammoths (I).

The artistic design on plaquette 2 is difficult to interpret, despite its completeness. There are about 400 intersecting incisions on the engraved surfaces of the two fragments. The straight parallel set of incisions (green and blue lines) perpendicular to each other are the more abundant type of design and the first set of incisions to have been engraved (Fig 10C and 10D). The more obvious and visible lines, however, are the circular lines concentrically defining the surface of plaquette 2 (Fig 10E). While parallel straight lines, intersecting at a right angle (blue and green lines) are common in Palaeolithic art [16,81,82] spiralling lines are rare and, when present, they usually depict portions of zoomorphic representations. The impression of a circular concentric design can be observed on several plaquettes at the Cueva de Caldas (Asturias, Spain; [83]), where, however, this design is the result of the superposition of several different zoomorphic subjects (in the case of plaque 1042, for instance, it is the result of the superposition of three mammoths, a rhinoceros and an anthropomorphic image; [83]). Circular concentric lines are also commonly used to represent antlers in the illustration of ibexes [e.g. 84]. On plaquette 2 from Jersey, the concentric (orange) lines (Fig 10E) are difficult to interpret. It is possible they were used to depict several stylised zoomorphic representations, however, we were only able to tentatively suggest the full body of a bovid on its left profile (Fig 10G). It is also possible that the concentric line were purely abstract, although the only examples of abstract depiction of concentric lines we could find in the literature have been associated with the decoration of perforated bone discs (*rondelles*) dated to the Middle Magdalenian in France and Asturias [85]. Finally, it is also possible that the concentric incisions were produced to frame the central portion of the plaquette, where the majority of the last (red) lines have been engraved (Fig 10F). We were expecting that the final incisions to be produced (the red lines) could depict some identifiable design. We anticipated that the greater strength used to produce these incisions (it has been experimentally demonstrated that a greater strength of cut produce wider and deeper incisions on bone; [59]) was purposefully applied to produce visible and recognisable artistic representations. We were unable to unambiguously associate any of these incisions with an obvious zoomorphic, anthropogenic or landscape representations. However, a possible interpretation is that the composition of some of the ‘red’ incisions in the central portion of plaquette 2 might depict a human face in frontal orientation (Fig 10G), similarly to the Group ε from La Marche (Lussac-Les-Châteaux, Vienne, France; [31]). Of particular interest is the presence of a semicircle over a cross (Fig 10F–10G), which could represent a human left eye, however, we are not aware of any other representation of this type during the Magdalenian period. Similarly to Group ε Hands ε2, the human-like face was engraved among a combination of straight lines intersecting each other perpendicularly, which makes its interpretation more enigmatic. It is also possible that some of the ‘red’ lines at the periphery of the central area might depict some very simplified zoomorphic representation of a horse in its right profile and, similarly to Gönnersdorf (Germany; [27]), three mammoths, two on their left profile and one, much larger, on its left side (Fig 10H). The intricacy of the incisions may mask other details of zoomorphic parts. The potential anthropomorphic and zoomorphic representations on plaquette 2 from Les Varines appear imprecise and overall simplified in comparisons to other Magdalenian examples, possibly suggesting that they were produced by inexperienced engravers.

### Fragment LVE 3249

**Fig 11:**
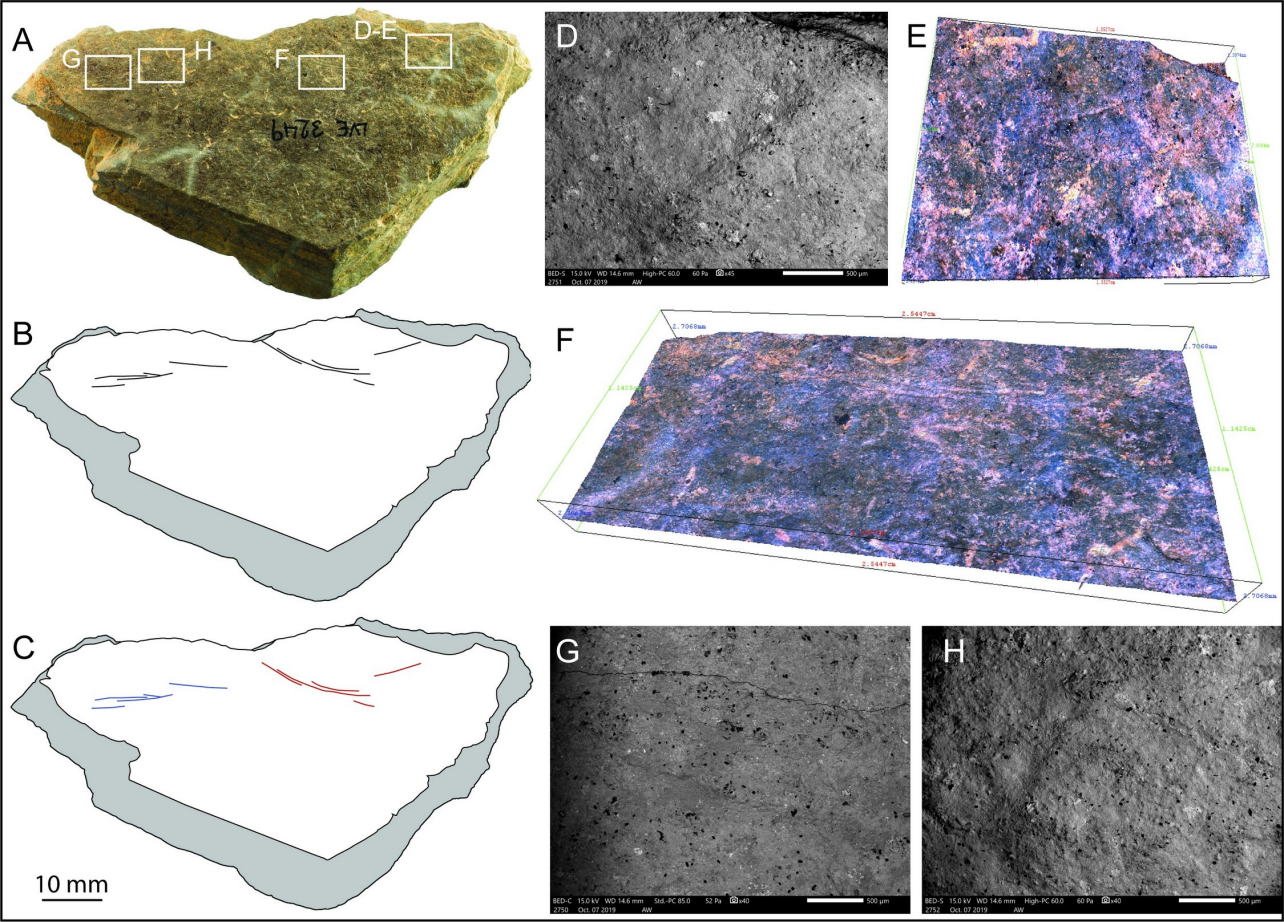
LVE 3249. A. Photo and B. drawing of LVE 3249. C. Drawing distinguishing straight incisions (blue lines) and sinuous incisions (red lines). D. SEM and Alicona 3D images of interrupted incisions at the edge of the stone fragment. F. Alicona 3D image of sinuous engravings and G-H SEM image of barely visible straight engravings.

LVE 3249, of triangular form, has a mid-dark grey/blue colour, and its degree of weathering and mineral composition make it very similar to plaquette 2. The engraved surface, however, appears less homogenous and coarser compared to plaquette 2 (Fig 11). Its two shorter sides are relatively smooth, representing probable natural edges of the original plaquette. The presence of two incisions on its top right (orientation according to Fig 11) interrupted by breakage, would suggest that it is only a fragment of a larger piece, however it does not display clear signs of deliberate fragmentation (Fig 11D and 11E).

Though large, LVE 3249 only displays 10 engravings. Five of these (blue lines in Fig 11C) are extremely shallow and barely visible, even at high magnification (Fig 11G & 11H). We were able to measure only two of them (Table 5). They are all single incisions. The remaining five incisions (red lines in Fig 11C–11F) at the opposite corner are rounded lines, slightly deeper, and more visible than the blue straight lines. They are formed by single incisions (two of them) and by double overlapping incisions (Table 5). Despite differences in the visibility of the two types of engravings, the micro-morphometric analyses of the incisions highlight the similarity in all profile parameters, suggesting that all marks were produced using the same tools, probably by the same engraver and in short succession.

**Table 5:** Average micro-morphometric values for the incisions on LVE 3249.

Stone fragment	Type of Incision	No Measured		Length (mm)	WIS (μm)	WIB (μm)	OA (°)	D (μm)
LVE 3249	BLUE	2–2	Mean =	**6.7**	**468.0**	**166.3**	**168.1**	**21.4**
			SD =	1.0	27.1	12.7	2.0	3.2
			Min =	5.9	448.9	157.3	166.7	19.1
			Max =	7.4	487.2	175.3	169.6	23.7
	ORANGE	5–5	Mean =	**9.1**	**443.2**	**145.9**	**154.6**	**34.4**
			SD =	2.6	136.9	51.2	7.0	10.9
			Min =	4.8	254.7	89.9	147.7	21.6
			Max =	11.2	623.5	226.5	164.1	44.1

No of measured incisions: the first number refer to how many lengths of incisions were measured, the second number refer to how many time profile parameters were measured. WIS: width at the surface; WIB width at the bottom of the cut; OA: opening angle of the incision; D: depth of the incision, ATI: angle of tool inclination.

There are too few engravings on LVE 3249 to suggest any clear design. The combination of straight lines and sinuous lines is reminiscent of the design observed on plaquette 1.

### Fragments LVE 9327

**Fig 12:**
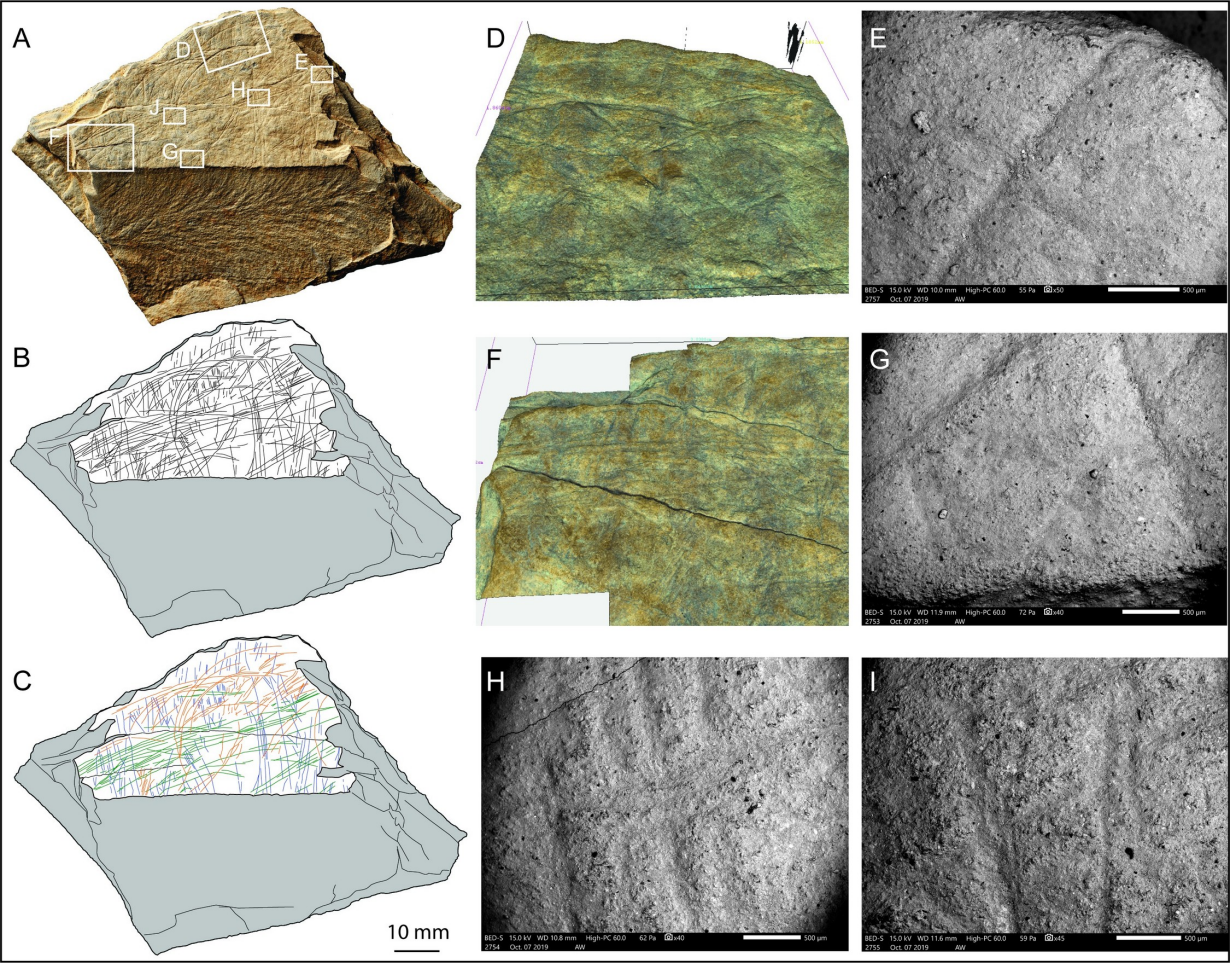
LVE 9327. A. Photo and B. drawing of LVE 9327. C. Drawing distinguishing straight incisions (blue and green lines) and sinuous incisions (red lines). D. Alicona 3D images showing the un-even engraved surface. E-G SEM and Alicona 3D images of interrupted incisions at different edges of the stone fragment. H. SEM image of straight ‘green’ incisions overlapping the straight ‘blue’ incisions. I. SEM image of the sinuous orange incisions overlapping the straight ‘green’ incisions.

LVE 9327 is a large flat stone of rectangular shape, which shows several irregular breakages. The engraving is preserved on a smaller triangular surface (approximately 62.8 x 35.0 mm), which is not completely flat but present some irregularities (Fig 12D). The fragment, pale brown in colour, is a piece of aplite/microgranite and has an appearance very similar to plaquette 1. The presence of several interrupted incisions at the edges of the engraved surface, suggests that LVE 9327 is a fragment of a larger plaquette (Fig 12E–12G).

Fragment LVE 9327 is marked by three types of incisions. The first incisions to have been produced are straight lines more or less parallel to each other and of irregular length (blue lines in Fig 12C). They are distributed on almost the totality of the engraved surface of the fragment. They are the thinner and shallower incisions on this plaquette and they are all been made by a single tool passage. Overlapping these incisions, there are longer sub-parallel lines (in green in Fig 12C and 12H) intersecting the previous incision at an almost 90-degree angle. These incisions are more concentrated in the mid portion of this stone fragment. They appear generally longer than the blue lines, as result of single long lines, or resorption of multiple incisions. They are the wider incisions on this plaquette fragment, as they are mainly produced by overlapping multiple tool passages. Finally, sinuous incisions with irregular orientation (in orange in Fig 12C) overlapped the other two types of incisions (Fig 12I). They cover the majority of the engraved surface of fragment LVE 9327, but are more concentrated toward an edge of the plaquette (upper portion in the orientation presented in Fig 12). Despite their sinuous shape, they are morphometrically similar to the ‘green’ incisions as they are also produced by a combination of single incision and incisions produced by overlapping multiple tool passages (Table 6).

**Table 6:** Average micro-morphometric values for the incisions on LVE 9327.

Stone fragment	Type of Incision	No Measured		Length (mm)	WIS (μm)	WIB (μm)	OA (°)	D (μm)
LVE 9327	BLUE	14/14	Mean =	**6.44**	**324.7**	**103.9**	**162.4**	**22.0**
			SD =	2.87	94.5	29.5	5.3	8.9
			Min =	2.14	180.0	48.2	151.9	7.9
			Max =	12.64	498.8	154.4	171.8	40.0
LVE 9327	GREEN	5/15	Mean =	**13.6**	**446.6**	**173.8**	**160.1**	**31.8**
			SD =	5.78	165.8	69.9	6.3	17.3
			Min =	7.65	214.1	43.4	144.0	9.5
			Max =	20.51	761.5	330.7	169.2	62.5
LVE 9327	ORANGE	13/20	Mean =	**11.51**	**397.1**	**149.1**	**153.4**	**38.6**
			SD =	5.49	151.2	61.5	8.2	25.9
			Min =	4.02	141.7	72.4	136.6	13.5
			Max =	18.11	725.7	325.1	166.97	115.7

No of measured incisions: the first number refer to how many lengths of incisions were measured, the second number refer to how many time profile parameters were measured. WIS: width at the surface; WIB width at the bottom of the cut; OA: opening angle of the incision; D: depth of the incision, ATI: angle of tool inclination.

**Fig 13:**
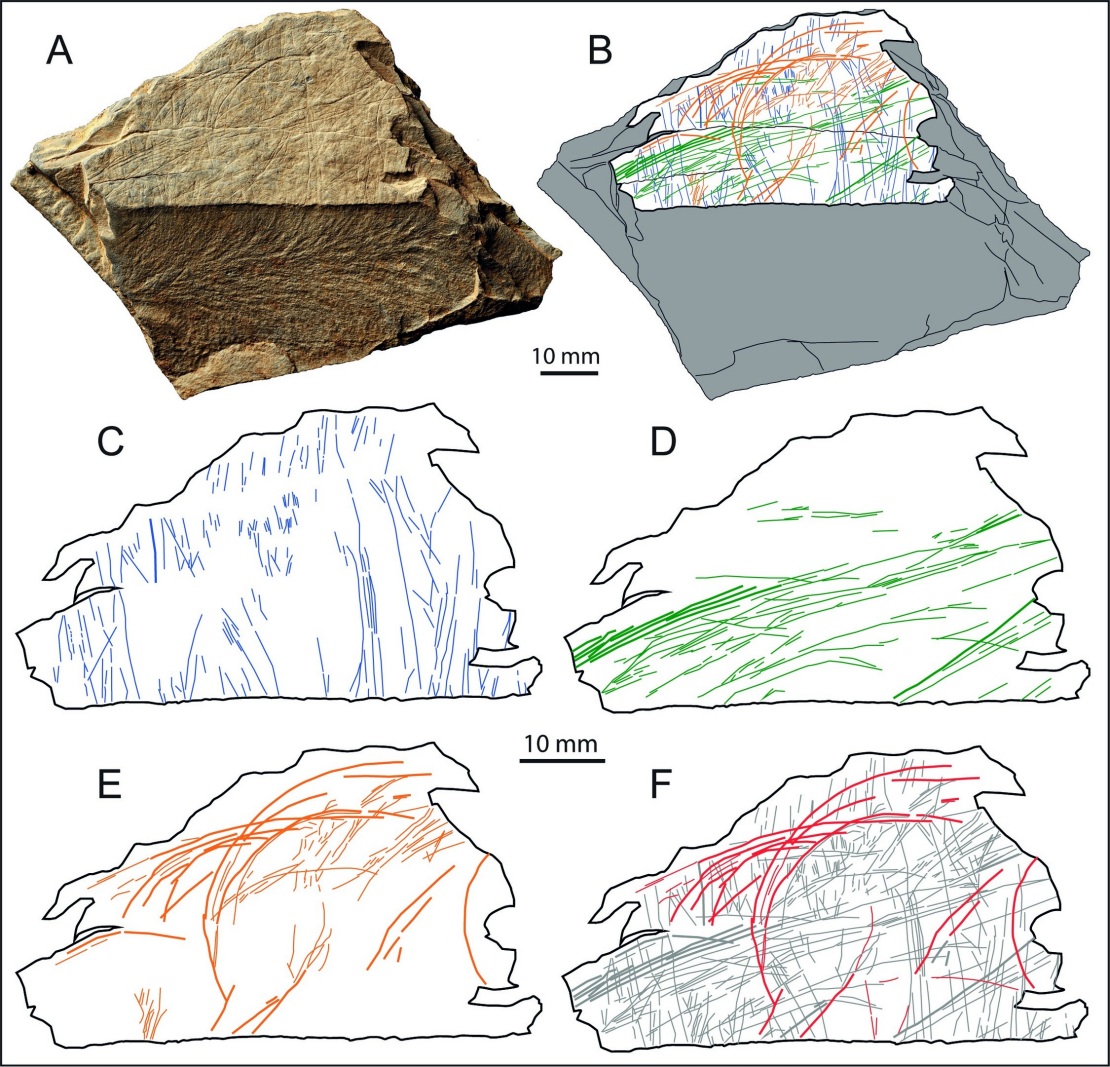
Fragment LVE 9327. A. Photo and B. drawing of fragment LVE 9327. Drawings separating the sequence of engraving: C. straight lines (blue lines) and D. straight lines (green lines) perpendicular to the previous ones. E. Sinuous, semi-circular incisions (orange lines), the last to have been engraved. F. tentative interpretation (red lines) of the back, hindquarter and tail of a horse.

There are about 400 intersecting incisions on the engraved surfaces of LVE 9327. As in the previous plaquettes and fragments of plaquettes at Les Varines, the parallel lines (green and blue) perpendicular to each other are the more abundant type of incisions and the first to have been produced (Fig 13C and 13D). In this case, however, the green lines are morphometrically more similar to the sinuous (orange) incisions. The sequence of engraving appears to have followed an organised chronological order (the three types of incisions overlap each other always in the same order: blue, green and orange), to produce an overall representation of abstract nature. The long sub-parallel incisions (green lines in Fig 13) are reminiscent of engravings described as ‘rivers’ observed on a stone block from the late Magdalenian site of Abauntz Cave (Navarra, Spain; [18]). The design depicted on this Spanish stone has been interpreted as a Palaeolithic map, due to the combination of incisions interpreted as ‘rivers’, ‘mountains or relief’, ‘flooded land’, ‘passes’ and ‘paths’. The fragmentary nature of LVE 9327 does not allow a clear interpretation of the engraved design, although we could suggest the long green lines could be interpreted as ‘river’ and the sinuous lines as ‘relief’. The sinuous lines, however, are more reminiscent of engravings representing horse backs and hindquarters (Fig 13F). Similar designs have been observed at Gönnersdorf [27], at Étiolles [39] and numerous other Magdalenian sites. It has in fact been recognised that horse is represented in more than three out of four sites with great constancy throughout the Upper Palaeolithic in all regions of Europe [86]. Therefore, it is possible that the curved lines on fragment LVE 9327 may depict the rear portion of a horse on its right side (Fig 13F). The elongated position of the horsetail could further suggest the illustration of a running horse [87]. However, the design represented by the orange lines on LVE 9327 is not complete and it is partially masked by others incisions, therefore this interpretation must remain tentative.

### LVE 7979

**Fig 14:**
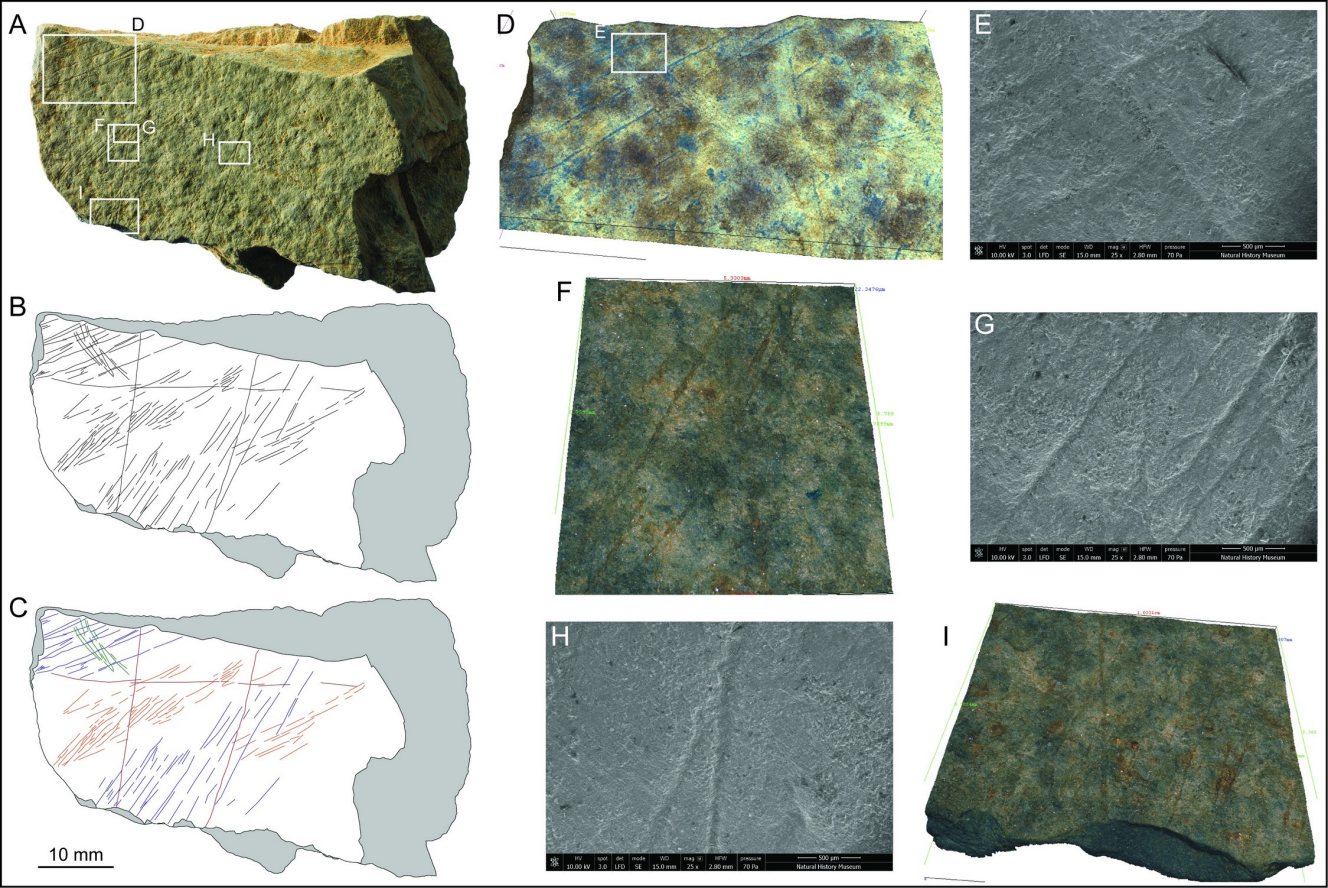
LVE 7979. A. Photo and B. drawing of LVE 7979. C. Drawing colour-coding the different types of incisions. D and I. Alicona 3D images of interrupted incisions at different edges of the fragment of plaquette. E. SEM image of sinuous ‘orange’ incisions overlapping the straight ‘green’ incisions. F-G. SEM images of ‘blue’ incisions. H. SEM images of the deep straight ‘grey’ incisions.

LVE 7979 is a fragment of stone plaquette of rectangular shape and of intermediate dimensions among the engraved stone plaquette fragments of Les Varines. It does not appear to display any natural edges and several engravings have been interrupted by breakage (Fig 14D and 14I), suggesting this fragment is a broken portion of a larger engraved plaquette. Like all other plaquette fragments, LVE 7979 is an aplite, with a general macroscopic aspect very similar to plaquette 1.

The engravings on this plaquette consist of at least four differently orientated clusters of lines all formed by single incisions. We colour coded these lines according to orientation, dimensions, and sequence of production as blue, green, orange and red lines. A first group of lines (blue lines in Fig 14C) is composed of two clusters of medium length oblique lines running more or less parallel to each other. They are located near the top left edge and the lower edge of the plaquette (according the orientation of LVE 7979 in Fig 14). Because of their location, most of these incisions are interrupted by the breakage (Fig 14D and 14I). These incisions are generally regularly spaced and sub-parallel. They appear to be overlapped by all other types of incision, suggesting they were the first to have been produced. A set of slightly more sinuous incision (green lines in Fig 14C) overlap, at a roughly perpendicular angle, this first set of ‘blue’ incisions (Fig 14D and 14E). Two further clusters of incisions (orange lines in Fig 14D) are distributed between the clusters of ‘blue’ lines and they are organised in a ‘fan-shape’ design rather than in series of sub-parallel lines. As with the long ‘blue’ lines, the ‘orange’ engravings are generally formed by short incisions, the result of interruptions and resumptions of successive incisions. The ‘orange’ incisions overlap the ‘blue’ incisions where these two sets meet in the central-right portion of the plaquette. As the ‘green’ and ‘orange’ lines do not overlap each other, we cannot determine in which order they were produced. Finally, nine straight incisions run the entire length of the plaquette, two from top to bottom, and one across from left to right. The line across (left to right) is not complete, but the result of interruptions and resumptions. These lines (in red in Fig 14C), with the exclusion of the ‘green’ lines which are not in contact with these incisions, overlap all other cluster of incisions, suggesting they were the last to have been made.

Regardless of their orientation and length, profile analyses suggest that the incisions were made by similar tools, if not the same tool (WIS and OA are very similar for all types of incisions; Table 7). The depth of incision is also regular for all engravings, possibly suggesting that there was no intention to make a single cluster of lines or a design more visually apparent (cf. deeper and wider incisions) than the other clusters. It is however possible, that any depth differences could have been faded by a weak generic polish, probably post-depositional, which has also obscured any possible traces of surface preparation.

**Table 7:** Average micro-morphometric values for the incisions on LVE 7979.

Stone fragment	Type of Incision	No Measured		Length (mm)	WIS (μm)	WIB (μm)	OA (°)	D (μm)
LVE 7979	BLUE	11–23	Mean =	**7.4**	**260.9**	**78.3**	**165.7**	**13.9**
			SD =	4.4	79.4	25.5	4.4	6.0
			Min =	1.9	161.7	43.9	158.7	5.3
			Max =	15.9	415.1	143.5	172.3	26.2
	GREEN	8–8	Mean =	**5.2**	**272.4**	**91.9**	**168.7**	**10.5**
			SD =	2.0	76.4	52.5	3.9	3.7
			Min =	3.4	184.8	53.1	162.9	5.5
			Max =	9.6	391.7	216.0	174.5	14.4
	ORANGE	15–15	Mean =	**4.0**	**237.5**	**71.1**	**161.9**	**15.9**
			SD =	2.1	66.3	21.8	5.1	6.2
			Min =	1.3	153.9	44.7	152.2	9.1
			Max =	10.1	391.9	120.8	169.2	29.5
	RED	5–8	Mean =	**21.2**	**264.2**	**81.5**	**164.2**	**15.4**
			SD =	6.0	82.3	45.6	5.2	6.2
			Min =	11.7	179.8	51.2	153.3	9.5
			Max =	28.4	452.2	190.5	168.9	28.4

No of measured incisions: the first number refer to how many lengths of incisions were measured, the second number refer to how many time profile parameters were measured. WIS: width at the surface; WIB width at the bottom of the cut; OA: opening angle of the incision; D: depth of the incision, ATI: angle of tool inclination.

**Fig 15:**
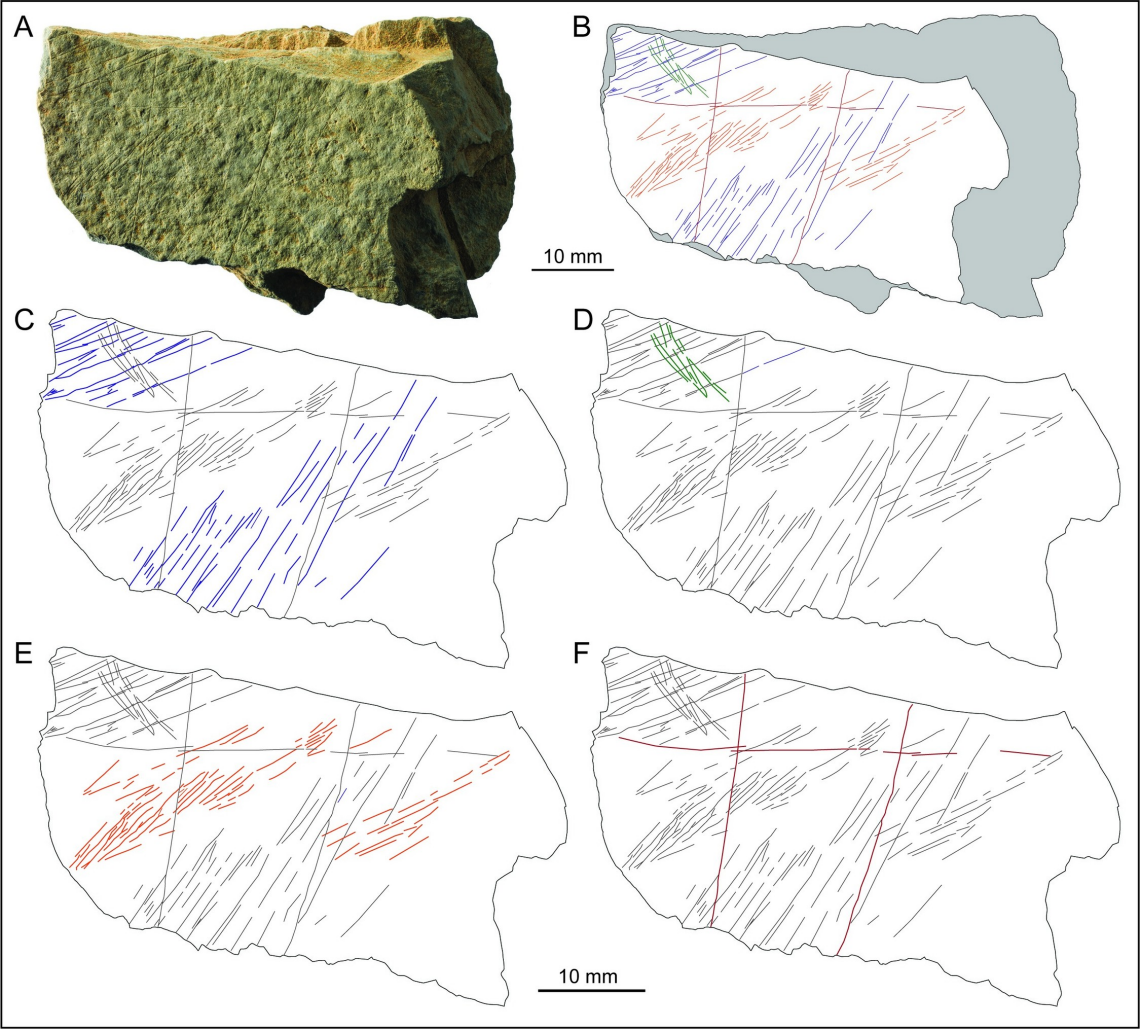
Fragment LVE 7979. A. Photo and B. drawing of fragment LVE 7979. Drawings separating the sequence of engraving: C. semi-parallel lines (blue lines), possibly the first to have been made and D. superimposing semi-straight lines (green lines) perpendicular to the previous ones. E. clusters of sinuous incisions (orange lines) and F. straight long crossing lines (red lines), probably the last one to have been engraved.

There are about 200 incisions on plaquette LVE 7979, forming an artistic design that shares some similarities with the engraving on the other fragments of plaquettes (Fig 15). The combination of sub-parallel straight lines is a common pattern among the design observed at Les Varines. Although the overall results is of abstract nature, the technique used to produce the short ‘orange’ incisions organised in a similarly fan-shape (Fig 15E), is reminiscent of the techniques used to represent the manes of horses or the bellies of a range of animals in Magdalenian art [8,23,39]. However, no complete zoomorphic representations are certain on this fragment of stone plaquette. The straight long crossing lines on LVE 7979 (Fig 15F) seem to be unique at Les Varines, but a similar pattern has been also observed at Foz Do Medal site (Portugal; [36,37]).

### Fragments LVE 9458

**Fig 16:**
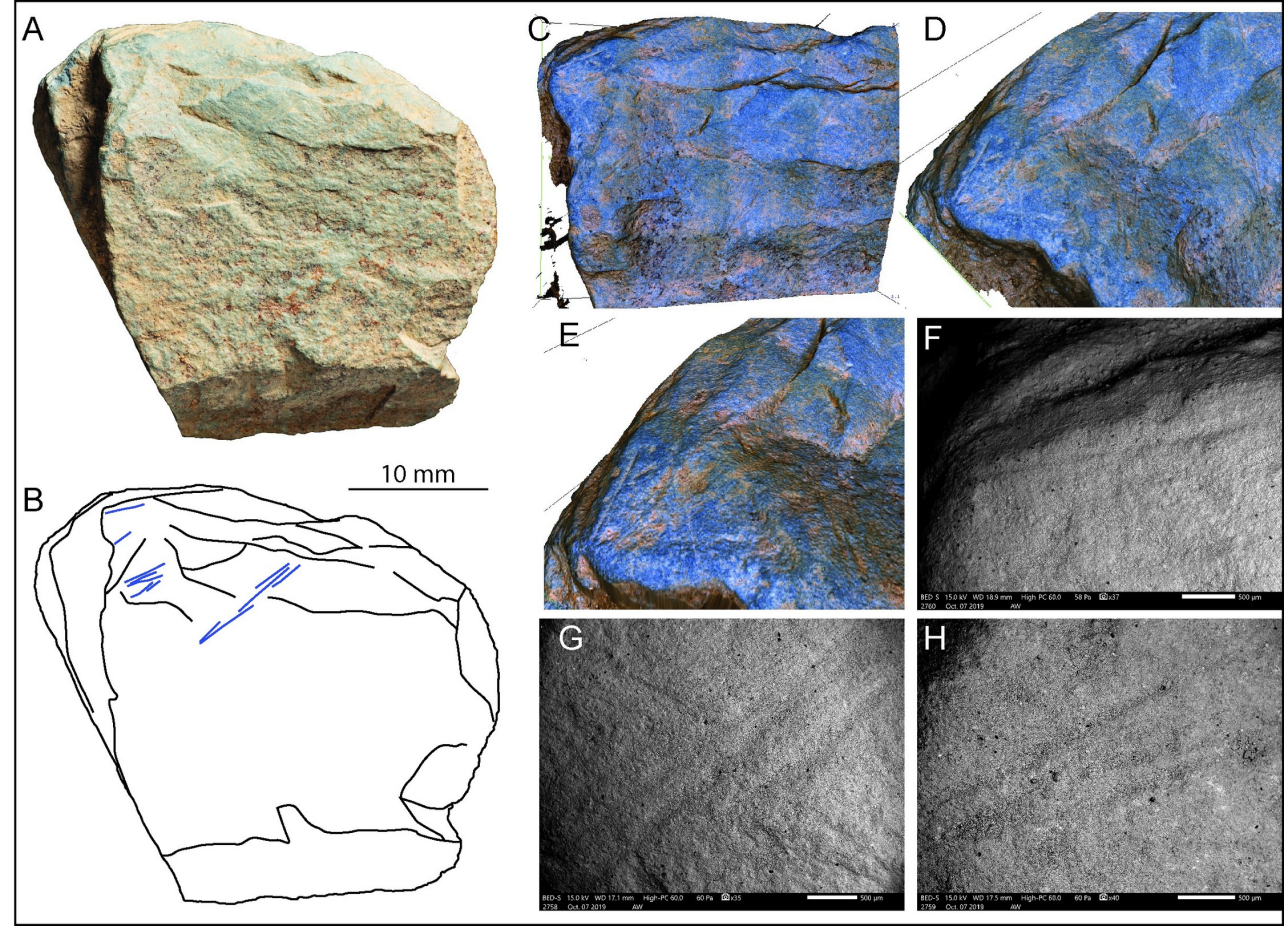
LVE 9458. A. Photo and B. drawing of LVE 9458. C-E. Alicona 3D images of interrupted incisions at the edge of the fragment of plaquette. Alicona 3D images also show the un-even surface on which the engraving was produced. F-H. SEM image of the incisions on LVE 9458.

LVE 9458 is a small fragment of a stone plaquette, which shows several irregular breakages similar to the one observed on LVE 9327. LVE 9458 is also an aplite and it is therefore possible that the two fragments (LVE 9458 and LVE 9327) originally belonged to the same stone plaquette. However, LVE 9458 appears to have a coarser (microgranite) aspect than LVE 9327. On LVE 9458, the few incisions (seven in total) are engraved on an uneven irregular surface, the only case observed among the ten engraved specimens of Les Varines (Fig 16).

All engravings are short, single passage incisions, generally thin and shallow (Table 8) and barely visible even at high magnification (Fig 16F–16H). Although we suggested that LVE 9327 and LVE 9458 could be fragments of the same plaquette, the micro-morphometric characteristics of the incisions on LVE 9458 are consistently different from the one recorded for any types of incisions measured on LVE 9327.

**Table 8:** Average micro-morphometric values for the incisions on LVE 9458.

Stone fragment	No Measured		Length (mm)	WIS (μm)	WIB (μm)	OA (°)	D (μm)
LVE 9458	7/7	Mean =	**4.3**	**261.1**	**99.2**	**160.0**	**18.6**
		SD =	3.1	68.5	44.9	5.0	7.7
		Min =	1.7	188.8	44.8	153.6	10.4
		Max =	10.2	369.3	178.7	167.6	29.5

No of measured incisions: the first number refer to how many lengths of incisions were measured, the second number refer to how many time profile parameters were measured. WIS: width at the surface; WIB width at the bottom of the cut; OA: opening angle of the incision; D: depth of the incision, ATI: angle of tool inclination.

There are too few engravings on LVE 9458 to suggest any clear design. Overall, these short incisions appear different from any pattern observed on the other engraved stone fragments as they are overall shorter and with more interruptions in their design. It is likely that these differences are due to the un-even nature of the surface on which the incisions were produced (Fig 16D and 16E).

### Fragment LVE 5322

**Fig 17:**
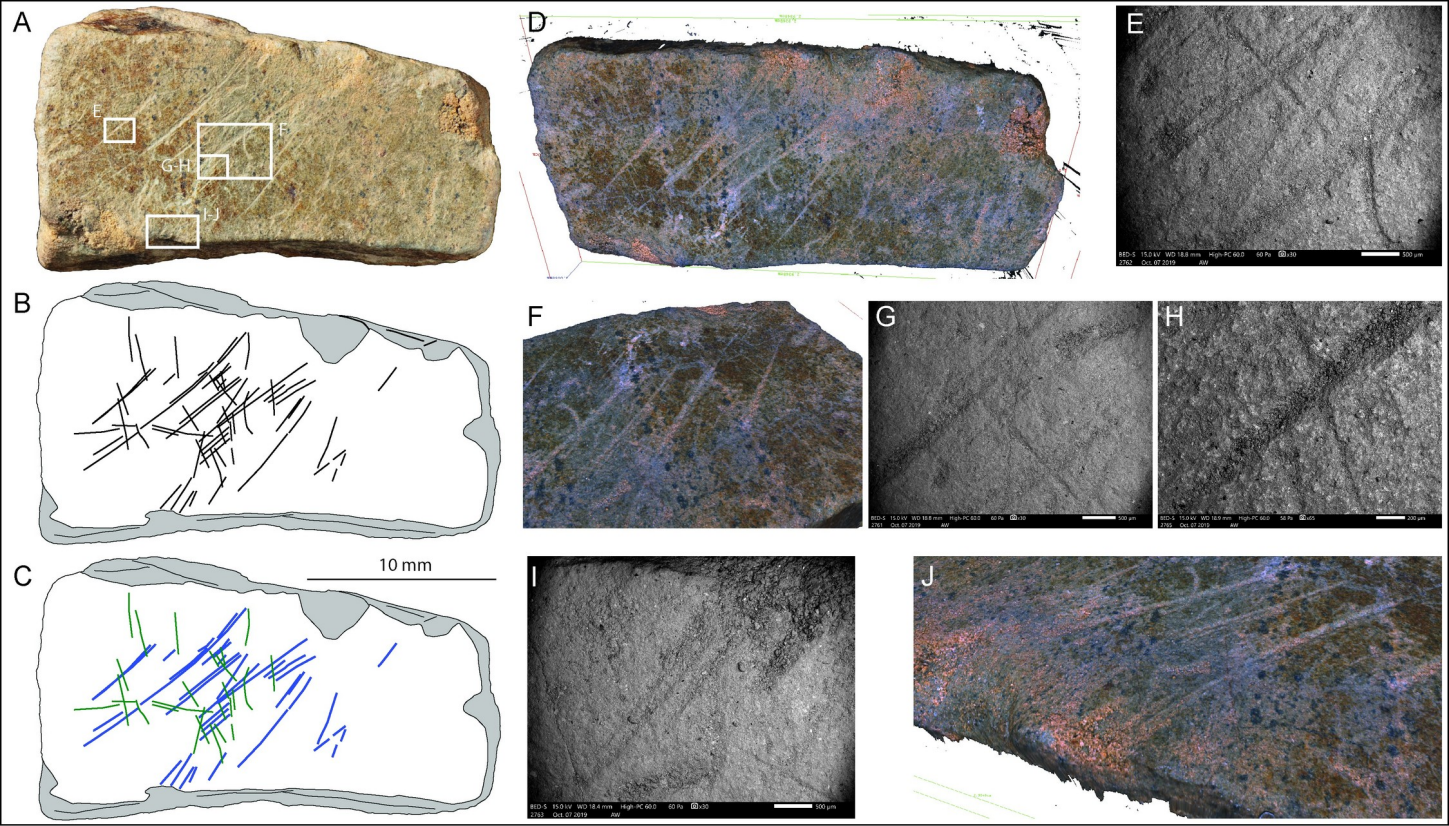
LVE5322. A. Photo and B. drawing of LVE 5322. C. Drawing colour-coding the different types of incisions. D Alicona 3D images of the entire engraved surface. E-H. SEM and 3D Alicona images of complex overlapping of incisions not visible at the naked eye. I-J SEM and 3D Alicona images of interrupted incisions at one of the edges of the stone fragment.

LVE 5322 is a small fragment which we were unable to refit to any of the other fragments uncovered (Fig 17). It has a macroscopic aspect similar to the fragments forming plaquette 1, so we cannot exclude that it was part of this larger artefact. One of the shorter sides is relatively smooth and very regular, representing a probable natural edge of the original plaquette. The fragment does not display clear signs of deliberate breakage.

Some incisions appear interrupted by the edges of the plaquette (Fig 17I and 17J), confirming that this stone fragment was probably part of a larger stone plaquette. Two sets of straight incisions are visible on the engraved surface: deeper and longer sub-parallel incisions (in blue in Fig 17) and shallower and shorter incisions (in green in Fig 17) intersecting the deeper ones at an angle of approximate 90° (Fig 17E–17H). Although shallower and less visible, the green incisions overlap the blue incisions when these two sets of engraving meet (Fig 17G and 17H), suggesting the blue incisions were the first to have been engraved (Table 9).

**Table 9:** Average micro-morphometric values for the incisions on LVE 5322.

Stone fragment	Type of Incision	No Measured		Length (mm)	WIS (μm)	WIB (μm)	OA (°)	D (μm)
LVE 5322	BLUE	9–9	Mean =	**6.7**	**296.9**	**107.3**	**158.8**	**22.9**
			SD =	2.3	72.1	29.3	6.9	13.0
			Min =	3.1	212.9	68.7	147.4	11.7
			Max =	9.7	452.8	155.7	170.4	52.7
	GREEN	4–4	Mean =	**2.5**	**208.4**	**72.2**	**165.7**	**11.1**
			SD =	0.7	54.2	25.6	2.1	2.9
			Min =	1.8	151.0	46.4	163.1	7.0
			Max =	3.6	269.6	101.4	167.6	13.3

No of measured incisions: the first number refer to how many lengths of incisions were measured, the second number refer to how many time profile parameters were measured. WIS: width at the surface; WIB width at the bottom of the cut; OA: opening angle of the incision; D: depth of the incision, ATI: angle of tool inclination.

There are too few engravings on LVE 5322 to recognise any clear design. However, the combination of straight sub-parallel incisions crossing at a 90-degree angle has been observed on the other plaquette fragments found at Les Varines, suggesting this was probably a recurrent abstract design at this Magdalenian site.

### LVE 4700

**Fig 18:**
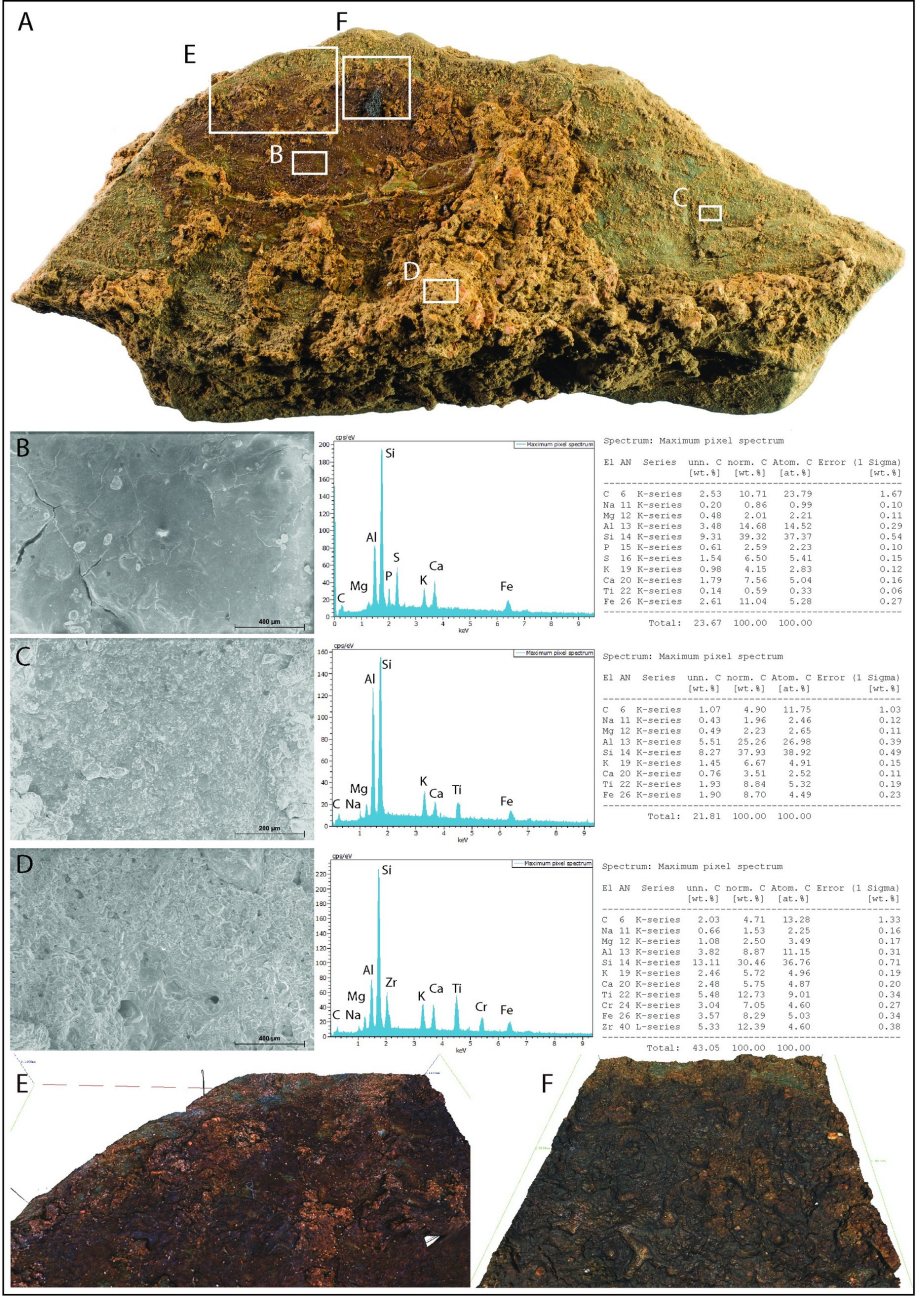
LVE 4700. A. Photo of fragment LVE 4700. SEM images and EDX analyses of selected areas on the stone fragment: (A) stained surface, (B) un-stained surface and (D) sediment still adherent on the surface of LVE 4700. E-F. 3D Alicona images of the stained area.

LVE 4700 is an unmodified plaquette, measuring 108x58x17mm, which appears to have been cleft in two horizontally. This action probably also detached a large flake (LVE 4469), measuring 54x33x6mm, which refits to the reverse face of this plaquette. This piece has a rougher surface but share the same rock type than all other engraved plaquettes (Fig 18).

LVE 4700 is not engraved, but presents a large stain (about 45x23mm) on its flat surface of a reddish colour (Fig 18A, 18E and 18F). Microscopically, the stained surface area appears smooth, coated by some substance probably liquid in its original form which dried out (this can be identified by the presence of cracks on the smooth surface, Fig 18B). This area (B) also has an elemental composition slightly richer in iron. SEM images of the un-stained stone surface free of sediment (Fig 18C) and of the un-stained surface, but still covered by adhering sediment (Fig 18D) appear courser and less even than the stained area. Because of its morphological and elemental characteristics, we originally hypothesised that the red staining might be a colourant (ochre?), possibly used to enhance the appearance of the engravings. However, the pigmentation seems to coat some areas of the stone covered by sediment, as visible on Alicona images (Fig 18E and 18F). This would suggest that the stone was possibly at least partially covered by sediment when came into contact with the colourant. It is possible that drops from an ochre-rich liquid substance may have fall on this stone during application on another plaquette (similarly to what has been reported by Roebroeks and co-authors [88]), although at the moment there are no unequivocal evidence that such activity was taking place at Les Varines.

## Discussion

The ten engraved stone fragments at Les Varines share common features. They were all made of fine-grained aplite/microgranite and were all engraved on a single surface. With the exception of fragment LVE 9458, which has an uneven surface engraved by few incisions, on all the other fragments the engravings were produced on a smooth flat surface. None of the plaquettes display any unequivocal traces which might indicate surface preparation for the engravings (e.g. grinding/smoothing), though any such traces are likely to have been removed by post-depositional processes which have led to the development of generic weak polish across these objects. All are broken fragments of larger objects with the exception of LVE 9581A and B, which form an almost complete plaquette (plaquette 2). The similarities in the micro-morphometrics of the incisions, suggest that, despite the presence of different designs, the engravings were produced by the same type of stone tools (probably a burin). Burins are generally regarded to be an effective tool for engraving stone due to their robust point [89,90]. However specific experimental work at Gönnersdorf suggests also broken flakes or blades can be used to incise plaquettes [29]. These types of tool are all common components of the Les Varines assemblage.

It is unlikely that these incisions are the result of a simple technical gesture (e.g. use of the plaquette as cutting support/anvil). Firstly, all plaquette fragments are generally small. They could have been part of larger stone support, but the near-complete plaquette 2, with a flat surface of about 57 mm x 56 mm, would have been too small to be used as an efficient cutting support/anvil. Secondly, although the presence of sub-parallel incisions could be an argument in favour of the use of the plaquettes as simple cutting supports, the presence of semi-circular, or sinuous, often deeper and more visible incisions strongly support the hypothesis of a purposeful artistic decoration. Moreover, although macroscopically the design on each plaquette appears different and with its own specificities, the combinations and intersecting engravings were consistently produced according a chronological order or in ‘layers’ (Table 10).

**Table 10:** Sequence of ‘layers’ of incisions for each engraved plaquette fragments from Les Varines (Jersey).

Plaquettes	Fragment(s)	Layer 1 (Straight sub parallel incisions)	Layer 2 (Straight sub parallel incisions overlapping layer 1 at an angle of about 90 degrees)	Layer 3 (Sinuous, irregular, curbed and/or concentric incisions)	Layer 4 (Irregular incisions, more visible, possible representation)
Plaquette 1	LVE 4394, LVE 4395 LVE 4607	yes		yes	
Plaquette 2	LVE 9581 (A and B)	yes	yes	yes	yes
	LVE 3249	yes		yes	
	LVE 9327	yes	yes	yes	
	LVE 7979	yes	yes	yes	yes
	LVE 9458	yes			
	LVE 5322	yes	yes		

On all the plaquettes from Les Varines the first engravings (the first ‘layer’) to have been produced were clusters of straight generally thin and shallow sub-parallel incisions (colour-coded in blue in this paper). This first design can be overlapped by a second layer of equally straight, thin and shallow sub-parallel incisions. When this layer is present, the incisions generally overlap the previous layer at an angle of about 90 degrees (colour-coded in green in this paper). In the case of plaquette 1 and fragment LVE 3249, this layer is missing and the straight incisions are instead overlapped by sinuous, irregular and in one case (plaquette 2) concentric lines (colour-coded in orange in this paper). These sinuous incisions represent layer 3 in the chronological sequence of engravings in the case of plaquette 2 and fragments LVE 9327 and LVE 7979. These sinuous incisions are often deeper and wider, due to a combination of single and multiple stone tool passages. This technique of engraving can possibly be associated with the intention of making them more visible in comparisons to the underling straight incisions. Some of these incisions could be associated with some form of zoomorphic representation, although we were not able to unequivocally associate any of these incisions with specific designs (Figs 7, 9 and 12). A final (fourth) layer is only present on plaquette 2 and LVE 7979 (colour-coded in red in this paper). This includes even deeper incisions which could also be linked to some form of representation (zoomorphic, anthropomorphic, or landscape depiction).

Regardless of the design produced at Les Varines, the consistency with which the engraving was performed is remarkable. It is difficult to think of a mundane technical sequence that would require numerous examples of a single gesture, over which were layered three more sequences of repeated gestures, each taking a different form. This occurs across several plaquettes and is not repeated as we might expect if the plaquettes were routinely used as technical supports such as cutting boards. This evidence strongly suggests that the plaquettes at Les Varines were engraved by purposeful artistic decoration.

The chronological order can possibly be explained by an increased degree of complexity, where the first clusters of incisions to be produced (straight thin shallow incisions) were the simplest to be made, followed by sinuous lines and finally the deeper and possibly more representative incisions. This order of engraving means that any representational element would always be difficult to read amongst a complex palimpsest of lines. Particularly interesting is the engraving on plaquette 2, where the superimposed incisions could hide several zoomorphic representations of a bovid, a horse and three mammoths, as well as a possible human face in front aspect, though none of these potential engravings are unequivocal. On all the fragments of plaquette analysed at Les Varines, these potential anthropomorphic and zoomorphic representations appear imprecise and simplified in comparisons to other Magdalenian examples, supporting either the hypothesis these are chance arrangements amongst a system of representations, or that they were the product of inexperienced engravers.

Overall, the design of abstract lines and possible zoomorphic (complete or partial) representations engraved on the different pieces from Les Varines, fit well within the broader corpus of Magdalenian engraved plaquettes. Across Europe, plaquettes are engraved with animals, stylised humans and abstract signs. In general abstract ‘signs’, outnumber representational forms by five to one according to Vialou [91]. At Roc-la-Tour 1, the nearest site to Les Varines with numerous plaquettes, the figure is closer to eight to one [16]. While a range of different signs are found on plaquettes and throughout Magdalenian art more broadly, sub-parallel lines tend to be the most common form, often occurring in the hundreds. 215 of the 625 signs at Roc-du-Tour 1 are lines occurring in clusters of between 2 and 15 [16]. It should thus be no surprise then that, given the small number of plaquettes recovered from Les Varines, all show groups of lines. Indeed, indecipherable designs are common across the core Magdalenian geographical range.

Beyond the designs engraved upon them, the broader context of plaquette manufacture and discard throw light on the significance of art in the daily lives of Magdalenian groups. Plaquettes are a type of art with a complex life history, for which engraving often only reflects one stage in a complex sequence of selection of raw material, production and shaping of the support, engraving, use and breakage or disposal. The first stage in the production of these plaquettes was the procurement of the aplite on which the engravings were produced. Aplite is a form of microgranite and the geology of Jersey is dominated by ancient granites associated with the Cadomian orogeny (450–700 Ma). The incised rocks reported here are of a type (aplite/microgranite) that is common in the St Helier granitic complex in Jersey, and so it is probable that people sourced the raw material for the plaquettes locally (Fig 4A). No immediately local source is accessible today, but the geomorphology of the area has changed considerably since the occupation and any local may now be masked by colluviated deposits. The use of local sources for engraved plaquettes is typical for the Magdalenian. The slate used at Gönnersdorf was sourced between 50 and 100m away [92]; at Roc-La-Tour 1, material was collected between 65m and 1km away [16] and at Enlène around 200m away [93]. In all these cases, the nature of the rock is particularly suitable to produce plaquettes. The fine-grained nature of tock types such as slates or schists, and their tendency to fracture along planes created by foliation, allow the creation of flat surfaces ideal for engraving (e.g. Gönnersdorf, in Germany; Roc-La-Tour in, France; Foz do Medal terrace in Portugal; [16,36]). Other stones were also used: at La Marche, thin limestone slabs were engraved [31], though this stone too is relatively soft. At Les Varines, while the rock type is different, the material properties of the selected raw material are the same.

Preparation of the plaquettes previous to engraving, possibly through grinding of the flat surface to make it smoother, cannot be recognised at Les Varines. But it is likely that some form of preparation was adopted before the surfaces were engraved. The surface of plaquette 1, for instance, is particularly flat which might suggest further modification, such as polishing. Some plaquettes on other sites show evidence of surface grinding or polishing [e.g. 36,37], though this never seems to have been a very extensive practice. Occasional polishing at Gönnersdorf may be related to erasure of previous designs [15].

Experimental work at Gönnersdorf has stressed the ease of production of engravings using a stone tool on soft stone and indicates that with only a little practice it was possible to engrave large surface in relatively short time [15]. Plaquettes have been suggested to be the work of inexperienced individuals just starting to learn how to make art, possibly apprentices, with the implication that experienced adults actively taught younger and inexperienced children [e.g. 91]. This is difficult to demonstrate but has received growing support and critical analysis in recent years [94–96] and parallels research conducted into teaching practices surrounding flint knapping in the Magdalenian [e.g. 97,98]. In wider discussions of art, there is growing evidence to suggest child authorship of some finger flutings, based on the size of the finger markings [99–102]. Bednarik [99] has, for example, suggested that children were the likely authors of painted fingerprints evident on limestone blocks from the Magdalenian layers of Hohle Fels, based on their small size. It is increasingly apparent that a range of authors of art can be expected and this is perhaps especially true for plaquettes which are frequently found in a domestic setting and evidence a wide variety of artistic skill. The engravings at Les Varines are composed of simple sub-parallel lines that could conceivably be produced at any level of skill. These lines may have been a backdrop to figurative deposition, acting to ambiguate the final engravings, or they could represent something more abstract. For an abstract depiction of this nature, it is difficult to directly translate skill onto the depictions from Les Varines, with skill being more readily discernible in figurative depictions. However, the production of clusters of incisions by layers, from a simpler design of straight lines to more complex design of sinuous deeper incisions, could suggest that possible learning practices were in place. In this context, each plaquette could represent a learning exercise aiming to improve the engraver’s skill from the production of simpler to more complex designs.

### But is it art?

A number of authors have argued that art is not an appropriate term to use when describing Upper Palaeolithic depictions. The term has a particular intellectual history in the west, where art is seen to embody transcendental qualities of universal concern, and above all is conceived of as visual representations to be admired and consumed [103,104]. This is not the case with Palaeolithic depictions: the presence of images in deep, inaccessible parts of caves and the prevalence of overwriting, both in caves (e.g. the sanctuary at Les Trois Freres) and commonly amongst plaquettes, suggests that some depictions were not meant to be widely viewed [105]. Many lines of evidence suggest that plaquettes held only brief significance, perhaps only at the moment of engraving. Engraving soft stone creates a powder within the incisions that makes them visible [16,29]. This swiftly disperses, meaning that the engravings are only clearly visible at the moment of their making [16]. Ochre can be used to make individual engravings more visible, as for example at la Marche [106], but traces of this mineral could not be found in direct association with the engravings at Les Varines, suggesting these markings were only temporarily visible. The evidence from Les Varines also suggests that the engravings were fleetingly seen and only of temporary significance. The potentially representational elements of the Les Varines designs were always engraved last in the sequence. As a result the pale powder generated by the act of engraving made the design briefly visible, before it was hidden amongst the palimpsest of earlier geometric phases.

In this context, a depiction to be admired is not the final result; rather the production itself and the act of engraving is more meaningful than the object (the plaquette) that has been engraved. This could explain why the vast majority of Magdalenian plaquettes appear to have been intentionally broken, both by percussion and by heating, possibly as a way of making sure the design was no longer readable. There has been considerable debate as to whether broken plaquettes were intentionally fractured. It has been suggested that fragmentation was used to disguise the subject of the engraving or as part as a magical ritual [e.g. 107–110]. However Bosinski and Fischer [27] point out, as unengraved plaquettes were similarly fragmented, breakage might be a result of trampling or frost-fracturing or post-depositional movement. Fragmentation could be a natural result of using plaquettes in hearth structures. Rozoy and Rozoy [16] suggest that, following engraving, there was no concern with keeping plaquettes intact, and they may have been broken in the course of daily activities or subsequent use. However, they also suggest that on occasions certain plaquettes were intentionally broken following engraving due to the nature of the design. A plaquette depicting a cervid from Roc-La-Tour which was thick and thus difficult to break was damaged instead with the head being removed and thrown away (ibid.). Similarly at the Magdalenian site of Foz do Medal Terrace, Portugal, some fragmentation appears intentional, with heat implicated in some cases, and the re-using of some of the fragments produced [37,111]. Bahn [112] notes the difficulty in interpreting fragmentation patterns that could be caused by a number of sources, but suggests that intentional fragmentation in association with some aspect of performance is a viable explanation for at least some plaquettes. At Les Varines rounding of the edges of breaks suggest these are ancient, rather than due to more recent post-depositional processes; however, there is no evidence for percussion marks that might signal intentionality.

At Les Varines the plaquettes were found in close association with hearths and pits and an area of pavement. Similar locations for plaquette deposition have been noted at Gönnersdorf, where plaquettes are associated with paving that formed the floors of structures and pits within the structure. Association with ochre has also been noted at the site with many of the engraved plaquettes clustering within a horizon of ochre in concentration I [29]. At la Marche too, engraved plaquettes formed pavements and many were painted with pigment prior to engraving [106]. Unengraved plaquettes also seem to have served as containers or crucibles for pigment production or as palettes for pigment processing and application, as at Pincevent [113]. At Les Varines, plaquette LVE 7979 was recovered from the edge of a pit filled with ochre, suggesting similar connections pertained.

Engraved plaquettes and other art *mobilier* have had less attention in Palaeolithic studies in the past in comparison to cave art, and many of the tropes particular to cave art have been imported to the study of plaquettes [114]. However, there are important differences: some cave art was undoubtedly meant to be viewed; some of the more elaborate panels of Magdalenian art are relatively near the entrance, in larger chambers, and more elaborately decorated, as at Altamira. Plaquette art, though itself dependent on the play of light for visibility, is perhaps more akin in others of its features to the parietal art found in the depths of caves, Clottes’ ‘art of the dark’ [105]. Paintings and engravings found in inaccessible areas in the depth of caves, are likely, as in the shaft scene at Lascaux, to have been seen by very few [115]. Some of this art of the dark is subject to similar overwriting as the plaquettes, the Sanctuary at Les Trois Frères, for example. However, cave art is completely bound up with place; cave art cannot be removed from its location and its engraving and viewing is entirely related to experiential aspects and the spiritual significance of entering an entrance into the earth. Clottes, for example, has argued that the surfaces of caves might have been highly significant, construed as a meeting point between worlds, a place through which spirit animals emerged through the membrane of the cave wall, and perhaps a focus for spiritual practices and experiences [115–117]. While some parietal depictions are found in inhabited caves, in rockshelters with domestic debris or the ‘supersites’ of the Pyrenees, such as Isturitz, the more elaborate painted caves were often set apart from areas of day to day life: Clottes [118] calls these sanctuaries. While many of Clottes’ arguments relate to southern France, a similar separation of caves for habitation and caves for art has been noted in Britain at Creswell Crags [41].

By contrast the context of engraved plaquettes is overwhelmingly domestic. At Les Varines, as at other plaquette sites they are associated with pits and hearths and possible dwelling areas. There is also some evidence that they may occupy an opposed sphere to cave art. The contiguous caves of Les Trois Frères and Enlène in the Pyrenees have very different inventories: Enlène has 1150 engraved plaquettes, Les Trois Frères only a single plaquette, but is the richest cave for parietal art in the region [34].

Although similar in dimensions to other form of mobiliary art, stone plaquettes are also very different from these. While elements of Magdalenian art *mobilier*, in particular beads such as *contours découpés* and decorated tools were truly mobile, accompanying people across different sites and landscape activities, most engraved plaquettes were not [119]: in general raw material was local, plaquettes were engraved in domestic contexts and then broken and left where they were made. They do thus seem very much connected with place, though a domestic space, very different from the space of cave sanctuaries. Their role can perhaps be seen as analogous to that posited by Verpoorte [120] for Pavolvian clay animal and human figurines at Dolni Vestonice. As with the plaquettes, these clay figurines were locally produced, briefly (if at all) seen, and seemingly not moved beyond the context of their production. Verpoorte suggests that manufacture of these clay figurines was about creating relationships with place, through incorporating local clay in the form of animal bodies around hearths, the focus of domestic space. The plaquettes may have worked in a similar way, incorporating something that was momentarily important into the foundations or structure of domestic space as hearth supports and pavement [see also 114]. Incorporation of the plaquettes into the domestic space of Les Varines may have been a way of taking possession of a place, important for people on the very edge of the Magdalenian world.

## Conclusions

The engraved fragments of aplite microgranite from Les Varines represent the first evidence of Magdalenian engraved stone plaquettes found in the British Isles, seemingly predating the Magdalenian portable art, parietal engravings and bas-reliefs at Creswell Crags, Derbyshire. The examples from Les Varines are an important extension of the northern and western signature of plaquette production, which remains rare in northern mainland France. The plaquettes from Les Varines bear many similarities to those found across the Magdalenian world in their material, themes, fleeting significance and fragmentation. The evidence from Les Varines suggests that the engravings were fleetingly seen and only of temporary significance and the process of engraving was the meaningful act rather than the viewing of the object (the plaquette) that had been engraved. The evidence from Les Varines also supports the hypothesis that plaquettes represent the art of place, but in contrast to cave art, this is the art of domestic space. The act of incorporating engravings momentarily charged with significance into the floors of structures and workplaces, into hearths and pits, was an important part of producing social and significant spaces.

## Supporting information

S1 Appendix (DOCX)
