# Supplementary material for: Artists on the edge of the world: An integrated approach to the study of Magdalenian engraved stone plaquettes from Jersey (Channel Islands)
Source: PLoS One. 2020 Aug 19;15(8):e0236875. doi: 10.1371/journal.pone.0236875 (PMC7446900; doi:10.1371/journal.pone.0236875)
Supplement: S1 Appendix — (DOCX) [file pone.0236875.s001.docx]

**Appendix 1. OSL Methods**

**OSL sample preparation**

Sample preparation and analysis was undertaken under safe light conditions at the luminescence laboratories at the School of Geography and Geosciences (SGG), University of St Andrews. Standard mineral preparation procedures as routinely used in OSL dating were used to obtain a 180-212µm HF-etched quartz fraction from each sample: including HCl and H_2_O_2_ treatments, heavy liquid density separation and HF etching (Spencer and Robinson, 2008). For samples LAM01, 02 & 06 quartz yields were low, and for these samples 212-250µm quartz fractions were also prepared. For LVE03 & 05 quartz yields were extremely low, and a only a few aliquots were prepared (for these samples, the ‘ages’ are only provided as a gauge, and should not be considered as quartz SAR OSL ages).

**Equivalent dose determinations**

OSL measurements were carried out using either a Risø TL/OSL DA-20 or Risø TL/OSL DA-15. Further details on this instrumentation can be found in Spencer and Robinson (2008). Initial experiments on preheat and dose recovery were conducted to determine that appropriate preheat temperature and to determine whether a hot bleach was required for each samples. De determinations were determined on sets of 24 to 108 aliquots using a single-aliquot regenerative dose (SAR) method (Murray and Wintle, 2000). For some samples, the SAR protocol was modified to include 1.) infra-red stimulation (IRSL) before OSL to optically remove unwanted feldspar signals; and 2.) an additional OSL bleach to remove luminescence due to thermal transfer (Spencer and Robinson, 2008; Morrocco et al., 2007).

Data reduction and De determinations were made in Luminescence Analyst and Excel. Aliquots were rejected from further analysis if they failed sensitivity checks (based on test dose response) and/or SAR acceptance criteria (% of aliquots accepted ranged from 30 to 80%). The distributions in equivalent dose values, for those aliquots which satisfied the SAR selection criteria, were examined using radial plots, statistical tests of normality, and CAM or

MAM age models.

**Dose rate determinations**

Concentrations of K, U, Th and Rb were measured directly using ICPMS in the CERSA analytical facilities at SGG. These data were used to determine infinite matrix doses for α, γ and β radiation, using the conversion factors of Adamiec and Aitken (1998), and grain-size attenuation factors of Mejdahl (1979). Present day moisture contents were used to gauge average moisture during sediment burial and were used to attenuate beta and gamma components. The cosmic radiation dose component was calculated following Prescott and Hutton (1994).

**Age determinations**

K, U and Th concentrations, the dose rates to HF-etched quartz these correspond to, and the stored doses from the Les Varines quartz are shown in table X, together with the quartz SAR OSL depositional ages.

**Additional References**

Mejdahl, V.  1979. Thermoluminescence dating: Beta-dose attenuation in quartz grains: *Archeometry*, 29 (1), 61-72.

Morrocco, S.M., Ballantyne, C.K., Spencer, J.Q.G. and Robinson, R.A.J.  2007. Age and significance of aeolian sediment reworking on high plateaux in the Scottish Highlands. *The Holocene*, 17 (3) 349-360.

Murray, A.S. and Wintle, A.G.  2000. Luminescence dating of quartz using an improved single-aliquot regenerative-dose protocol. *Radiation Measurements*, 32 (1), 57-73.

Prescott, J.R. and Hutton, J.T.  1994. Cosmic ray contributions to dose rates for luminescence and ESR dating: Large depths and long-term time variations. *Radiation Measurements*, 23 (2), 497-500.

Spencer, J.Q.G. and Robinson, R.A.J.  2008. Dating intramontane alluvial deposits from NW Argentina using luminescence techniques: Problems and potential. *Geomorphology,* 98, 144-155
